# Identification of reference microRNAs in skeletal muscle of a canine model of Duchenne muscular dystrophy

**DOI:** 10.12688/wellcomeopenres.22481.2

**Published:** 2024-11-20

**Authors:** Dominique O. Riddell, John C.W. Hildyard, Rachel C.M. Harron, Dominic J. Wells, Richard J. Piercy

**Affiliations:** 1 Department of Clinical Science and Services, Comparative Neuromuscular Diseases Laboratory, Royal Veterinary College, London, NW1 0TU, UK

**Keywords:** MicroRNA, DE50-MD, DMD, dog model, reference genes, RT-qPCR

## Abstract

**Background:**

Duchenne muscular dystrophy (DMD) is a fatal muscle wasting disease caused by mutations in the dystrophin gene. DE50-MD dogs are an animal model of DMD used as a final translational model for evaluation of promising treatments. MicroRNA (miR) expressions in the muscle of DE50-MD dogs represent potential biomarkers, but stable reference miRs must first be identified. The aim of this paper was to establish a panel of reference miRs for WT and DE50-MD dogs over a range of ages and muscle groups.

**Methods:**

RNA was extracted from WT and DE50-MD dog (N=6 per genotype) vastus lateralis muscle samples collected longitudinally at 3, 6, 9, 12, 15 and 18 months of age, and from muscles collected post-mortem (N=3 per genotype; cranial tibial, semimembranosus, lateral triceps and diaphragm). 87 RNAs were quantified in a subset of 6-month-old WT and DE50-MD muscles (N=4 per genotype) using the QIAcuity miFinder panel. GeNorm, BestKeeper and Normfinder were used to identify a candidate panel of the 8 most stable small RNAs, which were then quantified in all RNA samples, alongside the commonly used reference RNA snRNA U6.

**Results:**

The most stable miRs of this subset were used to normalise quantities of dystromiRs miR-1, miR-133a and miR-206, and fibromiR miR-214. MicroRNAs miR-191, let-7b, miR-125a and miR-15a were the most stable miRs tested, while snRNA U6 performed poorly. DystromiR expression, normalised to the geometric mean of the panel of reference miRs, was lower for miR-1 and miR-133a in DE50-MD compared to WT muscles, while miR-206 levels did not significantly differ between genotypes. FibromiR miR-214 was 2- to 4-fold higher in DE50-MD versus WT muscles.

**Conclusions:**

A normalisation factor derived from miR-191, let-7b, miR-125a and miR-15a is suitable for normalising miR expression data from WT and DE50-MD muscle over a range of ages and muscle types.

## Plain Language Summary

Duchenne muscular dystrophy (DMD) is a fatal muscle wasting disease caused by mutations in the dystrophin gene. DE50-MD dogs are a canine model of DMD that manifest the disease in a very similar way to human DMD patients. To further our understanding of DMD and to identify potential markers of an improvement in the condition following treatment, we aimed to quantify the expression of several microRNAs (miRs) in muscle samples. miRs are small RNA molecules that regulate the expression of genes, whose expression is often altered in DMD patients. To ensure reliable quantification, we must first identify miRs that are stably expressed in muscle to use as a normalisation factor that will account for any variation that may be introduced during the experimental process. We enrolled 6 healthy and 6 DE50-MD dogs and collected muscle samples at 3-monthly intervals from 3- to 18-months of age. RNA was extracted from these samples and a large panel of potential reference RNAs were quantified. We used 3 different algorithms to test the stability of each of these RNAs in our sample pool: GeNorm, BestKeeper and Normfinder. MicroRNAs miR-191, let-7b, miR-125a and miR-15a were the most stable miRs tested. These 4 miRs were used to normalise the amount of several miRs of interest to DMD. Expression of miRs of interest miR-1 and miR-133a, when normalised to the top 4 reference RNAs, were reduced in DE50-MD compared to healthy dogs, while miR-206 levels did not differ between groups, and miR-214 levels were increased. These results are consistent with findings in human DMD patients. We conclude that normalisation to expression of miRs-191, let-7b, miR-125a and miR-15a is suitable for reliable quantification of miR data from DE50-MD and healthy dog muscle over a range of ages.

## Introduction

Identifying biomarkers of pathology is a vital component of the study of neuromuscular disorders. They can be used to assess disease severity, progression, response to a therapeutic intervention, and can contribute to our knowledge of pathophysiological mechanisms. microRNAs (miRs) are a class of small (approximately 22 nucleotides in length) RNAs involved in gene expression regulation ( Ambros, 2004), that are a potential source of such biomarkers. In the case of the fatal, muscle-wasting neuromuscular disease Duchenne muscular dystrophy (DMD), alterations in miR expression in affected muscle and in the circulation have been associated with the phenotype ( Lee *et al.*, 2022). DMD is a genetic disorder caused by mutations in the dystrophin gene that result in an absence of a functional dystrophin protein, a protein critical for normal muscle function, stability and signalling ( Duan *et al.*, 2021). DMD patients undergo repeated muscle damage and degeneration, followed by regeneration, that ultimately results in failure to repair and maintain sufficient functional muscle ( Luz *et al.*, 2002).

One group of miRs whose expression is significantly altered in the tissues of dystrophic compared to healthy patients are miR-1, miR-133a and miR-206, collectively referred to as the dystromiRs for their specific expression in muscle cells and their roles in muscle maintenance ( Cacchiarelli *et al.*, 2011). These 3 dystromiRs are elevated in the serum of both DMD patients and the *mdx* mouse model of the disease, however both miR-1 and miR-133a are typically lower or unaltered in dystrophic compared to healthy muscle tissue, while miR-206 shows either no difference or mild upregulation ( Israeli *et al.*, 2016; Roberts *et al.*, 2012; Zaharieva *et al.*, 2013). Ambulant DMD patients have higher circulating dystromiR concentrations than non-ambulant patients who have a greater degree of muscle atrophy and reduced activity levels ( Almeida-Becerril *et al.*, 2022). Similarly, patients on a daily steroid regimen that supresses the immune response hence promoting muscle fibre integrity and preserving muscle mass, have higher serum dystromiRs than untreated patients ( Zaharieva *et al.*, 2013). Such findings suggest that serum dystromiR concentrations are associated with muscle mass and might be influenced by activity. This conclusion is, however, in contrast with another study that found that the concentration of circulating dystromiRs paralleled disease severity (based on North Star Ambulatory Assessment) ( Cacchiarelli *et al.*, 2011). It thus currently remains unclear whether the increased circulating dystromiRs in dystrophic patients result purely from damaged and leaking muscle membranes, or altered expression associated with muscle pathology, or a combination of the two ( Almeida-Becerril *et al.*, 2022; Roberts *et al.*, 2012; Zaharieva *et al.*, 2013).

Another group of miRs that are of interest to DMD are those involved in muscle fibrosis. Fibrosis is characterised by the excess deposition of extracellular matrix (ECM) proteins and is a prominent feature of pathology in dystrophic muscle ( Zhou & Lu, 2010). The repeated cycles of muscle degeneration and regeneration in DMD result in chronic inflammation associated with the persistent production of profibrotic cytokines and consequent synthesis and accumulation of ECM proteins such as collagens and fibronectins within the tissue ( Desguerre *et al.*, 2009). The expression of several miRs is associated with fibrosis in DMD muscle, including miR-21, miR-29, miR-199a-5p and miR-214-3p ( Arrighi *et al.*, 2021; Morgoulis *et al.*, 2019; Zanotti *et al.*, 2015). A study using DMD patient muscle biopsies showed that, of these fibromiRs, miR-214-3p most strongly correlated with muscle fibrotic content and was the most potent regulator of fibro-adipogenic progenitor cells in culture ( Arrighi *et al.*, 2021).

For the purposes of identifying novel biomarkers, furthering our understanding of disease pathogenesis and in particular for translational preclinical drug trials, substantial efforts have been put into the development and characterisation of large animal models of DMD. Both pig ( Klymiuk *et al.*, 2013) and dog models ( Sharp *et al.*, 1992; Walmsley *et al.*, 2010) of DMD have been developed, that more closely represent the disease phenotype seen in DMD patients, compared to the commonly used *mdx* mouse ( Wells, 2018). While mouse models have the benefit of allowing us to study large numbers of animals for high-powered studies, they show minimal clinical signs of disease and live near-normal life-spans ( Wells, 2018). The DE50-MD dog model of DMD is a beagle-cross breed with a point mutation in intron 50 of the dystrophin gene, that results in deletion of exon 50 in mature mRNA transcripts ( Walmsley *et al.*, 2010). The phenotype and progression of disease in this canine model closely parallels that of DMD patients before the point of loss of ambulation ( Hildyard *et al.*, 2022; Hornby *et al.*, 2021; Riddell *et al.*, 2024a; Riddell *et al.*, 2023; Riddell *et al.*, 2024a; Riddell *et al.*, 2021; Walmsley *et al.*, 2010). As seen in DMD patients, the mdx mouse and other dog models of DMD, the DE50-MD dog has dramatically elevated levels of dystromiRs in their serum ( Riddell *et al.*, 2021) and significant fibrosis in their skeletal muscles compared to wild-type (WT) age-matched dogs ( Hildyard *et al.*, 2022). To contribute to the understanding of dystromiRs and fibromiRs in the pathogenesis of DMD and potentially to add to the panel of disease biomarkers for this animal model, it would be of value to quantify their expression in muscle of DE50-MD compared to WT dogs. In particular, and in comparison with serum analysis, an advantage of quantifying miRs in muscle tissue is that muscle expression of the miRs is assessed without confounding factors of membrane integrity and muscle mass; moreover, miRs can be assessed in the same tissue for which histopathological assessment is possible, feasibly then providing direct mechanistic insight into the roles of miRs within DMD-affected muscle. However, muscle biopsy is relatively invasive in comparison with blood sampling, and typically only a small biopsy sample of a single muscle is obtained and evaluated. In contrast, serum analysis enables more body-wide assessment. Consequently, there is a place for both muscle and serum analysis. Large animal models of DMD such as the DE50-MD canine model enable sampling of a wide range of muscle tissues at multiple timepoints, in animals with a phenotype more similar to that of DMD-affected humans than equivalent rodent models.

Quantification of miRs can be achieved using reverse transcription quantitative PCR (RT-qPCR), however, suitable, stably expressed reference RNAs must first be established. The use of reference RNAs can account for variation between samples that might be introduced during sample processing, RNA extraction, sample storage, or the RT-qPCR assay procedure ( Bustin & Nolan, 2004). Reference genes determined for conventional gene expression studies are rarely suitable, as miRs are smaller, require specialised isolation and reverse transcription approaches, and are moreover often measured within samples not considered transcriptionally active (such as serum or plasma): references should accordingly be miRs or other small RNAs. When comparing samples from both DE50-MD and WT control dogs, the skeletal muscle transcriptional milieu is likely to differ considerably in dystrophic muscle that is undergoing myofibre necrosis, degeneration, regeneration and associated pathology, compared to the relatively stable state of healthy muscle ( Hildyard *et al.*, 2019). Alterations in dystrophic dog muscle miR expression have previously been demonstrated in the Golden Retriever muscular dystrophy (GRMD) model in both the biceps femoris and the relatively spared, cranial sartorius muscles ( Nghiem *et al.*, 2013; Sanson *et al.*, 2020). Both studies analysed muscle taken at a single age, but there could be differences in gene expression dynamics between dystrophic dogs of different ages as the severity and phases of pathology evolve. Further, we can expect differences between muscle groups due to variation in the extent of pathology in one muscle group compared to another ( Hildyard *et al.*, 2018). Identification of RNAs that remain stable across genotypes, ages and muscle groups is thus essential, allowing accurate normalisation of miR expression data across these samples.

There is a lack of validated reference miRs in the literature ( Veryaskina *et al.*, 2022) and no consensus on the most appropriate reference miRs for dystrophic muscle, with previous studies normalising muscle dystromiRs to miR-93 ( Israeli *et al.*, 2016), miR-16 ( Roberts *et al.*, 2012), small nuclear RNA U6 (snRNA U6) ( Zaharieva *et al.*, 2013), or a combination of all 3 in the case of a study utilising the GRMD canine model of DMD ( Sanson *et al.,* 2020) Indeed, the most appropriate muscle reference miR/miRs will likely vary study to study, depending on the species, age and specific muscles analysed. snRNA U6, an approximately 100-nucleotide RNA located at the heart of the spliceosome ( Didychuk *et al.*, 2018), is the most commonly used reference RNA for miR normalisation. However, several studies reveal that U6 in fact has variable expression across species, disease states and cell types ( Jacobsen *et al.*, 2016; Peltier & Latham, 2008). We previously determined that miR-223 was stably and robustly expressed in DE50-MD and WT control serum samples, and we therefore used it as a suitable reference miR for quantifying serum expression of dystromiRs in this dog model ( Riddell *et al.*, 2021). However, evidence from other studies shows miR variation between tissue and cell types within species, emphasising the importance of establishing appropriate reference genes for each novel combination of species, disease and tissue of interest ( Peltier & Latham, 2008).

There are several algorithms available that aim to identify suitable reference genes/RNAs including geNorm, Bestkeeper and Normfinder ( Andersen *et al.*, 2004; Pfaffl *et al.*, 2004; Vandesompele *et al.*, 2002). geNorm and Bestkeeper use pairwise comparisons of the panel of potential reference targets in order to determine their stability, while Normfinder assesses the stability of individual candidates ( Hildyard *et al.*, 2019). geNorm measures the pairwise variation of a reference candidate to each other candidate within a sample, to give a stability value (M) that reflects the extent to which levels of that candidate vary from that of the others; by applying this approach iteratively, discarding the most variable candidate at each step, geNorm identifies the pair of candidates that vary in the most correlated fashion (the best pair). Bestkeeper calculates the geometric mean of the Cq values of every candidate gene within a sample to give a ‘whole panel’ normalisation factor (the Bestkeeper) and then performs pairwise comparisons of each candidate with this factor to give a Pearson correlation. Thus, like geNorm, Bestkeeper assesses which candidate best reflects the behaviour of the whole pool of candidates. In contrast, Normfinder calculates sample-to-sample variability for individual candidates. The Normfinder algorithm has the advantage of optional grouped analysis: a candidate might exhibit variation within or between groups that could be masked when all data is analysed together. Due to their different approaches, none of these mathematical methods is the gold standard; instead, using them in combination is a more robust method of reference target selection ( Veryaskina *et al.*, 2022).

The aim of this study was to identify stable reference miRs in skeletal muscle of the DE50-MD dog model of DMD and its WT littermates, across different ages and muscle groups. Once identified, these reference miRs could then be used to normalise quantification of the dystromiRs and other miRs of interest to DMD in the skeletal muscle of this disease model.

## Methods

### Animal husbandry

Dogs ( *Canis familiaris*) used in this study were from the DE50-MD colony, housed in a dedicated facility at the Royal Veterinary College (RVC), London. WT and DE50-MD dogs were housed together, typically in groups of 2–3 (groups determined according to dog temperament and hierarchies). Kennel size was 4.5 m ^2 ^to 7.5 m ^2^ and the Animal (Scientific Procedures) Act 1986 (ASPA) code of practice (Section 2) regarding the number of dogs per area of kennel size was followed at all times. Dogs were housed in indoor kennels (12-hour light/dark cycle, 15–24°C) with daily access to large outdoor paddocks (approximately 100 m ^2^). Dogs for this study were produced by breeding carrier female Beagles (RCC strain, Marshall Bioresources)-cross dogs, with WT male Beagles (RCC strain). At seven days of age, puppies were microchipped and cheek swabs were taken for preparation of DNA (GeneJET genomic DNA kit, #K0721, Thermofisher ^TM^) for genotyping. Polymerase chain reaction (PCR) was performed on a Veriti 96 Well Thermal Cycler (Applied Biosystems) using primers spanning the DE50-MD mutation site (splice donor site of dystrophin exon 50): Forward primer 5’-3’ sequence AGCTCTGATTGGAAGGTGGT; Reverse primer 5’-3’ sequence ACCTCAGTGTTGTGCTTTTGA. PCR products were sent for Sanger sequencing (SupremeRun, Eurofins Scientific) using the forward primer only. Other than a genotype of WT male or DE50-MD male, no additional exclusion criteria or randomisation strategies were used when recruiting dogs. All dogs that were not recruited to studies were rehomed. Staff and researchers involved in animal husbandry, data acquisition and data analysis were not blinded to genotype.

### Arrive guidelines

ARRIVE guidelines were followed at all times and an ARRIVE E10 checklist was completed (see Extended data ( Riddell *et al.*, 2024b)). The study was conducted within project license P9A1D1D6E (granted 11 June 2019) assigned under the Animal (Scientific Procedures) Act 1986, according to UK legislation and was approved by the Royal Veterinary College local Animal Welfare Ethical Review Body. Dogs were observed daily by animal technician staff and any concerns were reported to the Study director, Named Veterinary Surgeon and Named Animal Care and Welfare Officer. All efforts were made to minimise any animal suffering throughout the study. Humane endpoints were established before commencing the study: dehydration (unresolved by fluid treatment), lethargy/motor dysfunction, weight loss/dysphagia, dyspnoea, listless behaviour/demeanor or heart failure. None of the dogs included in this study reached humane end-points during the 18-month study period.

### Study population

In total, samples from 8 male DE50-MD and 9 male WT dogs were used in this study. This cohort of dogs was studied longitudinally from birth until 18 months of age as part of a comprehensive natural history study, further data from which is published elsewhere ( Hildyard *et al.*, 2022; Hornby *et al.*, 2021; Riddell *et al.*, 2021; Riddell *et al.*, 2022; Riddell *et al.*, 2023). The sample size for the natural history study was determined in order to give a robust estimation of the variance of the population, allowing sample size calculations to be performed for prospective studies. All 8 DE50-MD dogs and 3 of the WT dogs were humanely euthanised at 18 months of age (250 mg/kg sodium pentobarbital (Dolethal, Covetrus)). The remaining 6 WT dogs were rehomed.

### General anaesthesia

Muscle was biopsied routinely under general anaesthesia. Dogs were pre-medicated with intravenous methadone (0.2mg/kg, Comfortan, Dechra) via venous catheter, prior to induction of anaesthesia by intravenous propofol (1–4 mg/kg, PropoFlo Plus, Zoetis). Anaesthesia was maintained by sevoflurane (1.5–3.5%, SevoFlo, Zoetis) in oxygen via endotracheal tube using a Aestiva/5 anaesthesia machine (Datex-Ohmeda). During the procedure, dogs were given intravenous cefuroxime antibiotic (20 mg/kg, Zinacef, GSK) and carprofen for analgesia (2 mg/kg, Rimadyl, Pfizer). Following the procedure, post-operative carprofen (2 mg/kg, Rimadyl, Pfizer) was given orally, once daily for 3 days.

### Muscle sampling

**Figure 1.**
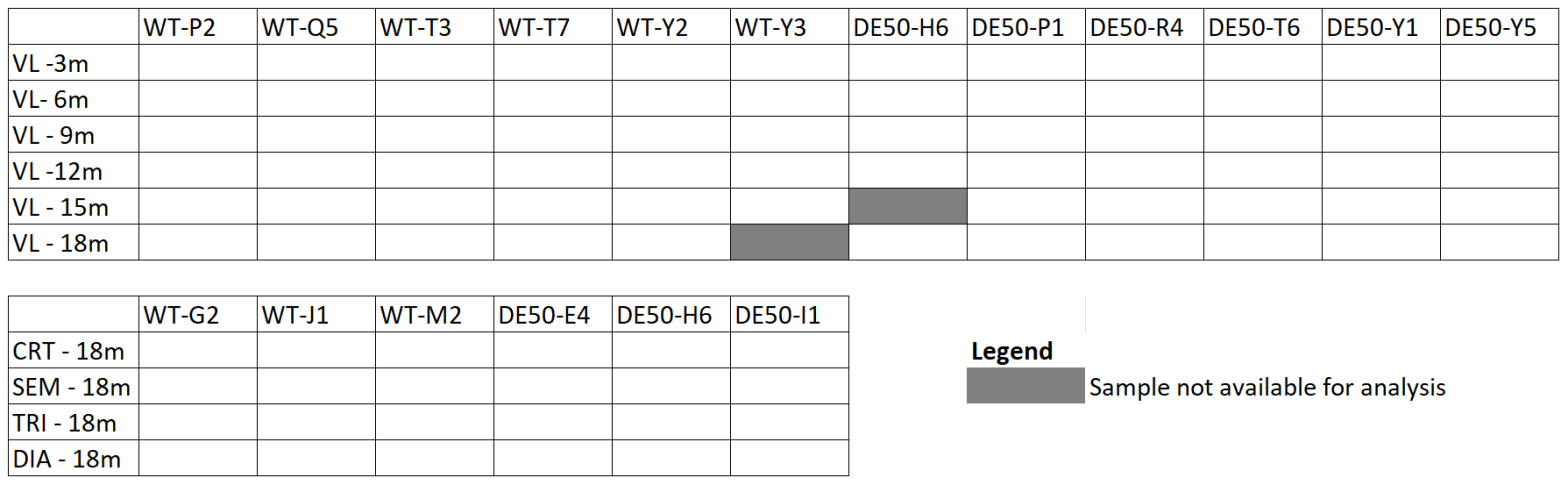
Samples used for RT-qPCR assays. Muscle samples (vastus lateralis (VL), cranial tibial (CRT), semimembranosus (SEM), lateral triceps (TRI) and diaphragm (DIA)), age of dogs at time of sampling in months (m) and dog ID (abbreviations beginning with WT are wild-type dogs and those beginning with DE50 are dystrophic DE50-MD dogs) for RT-qPCR assays. Boxes shaded in grey were not available for analysis.

Vastus lateralis (VL) samples (approximately 3x3x6mm) were collected by open-biopsy under general anaesthesia at 3-monthly intervals between 3 and 18 months of age (as described elsewhere ( Hildyard *et al.*, 2022)). Muscle samples were collected post-mortem from dogs that were euthanised at the end of the 18-month study period. Of these, 4 muscles were analysed: cranial tibial (CRT), semimembranosus (SEM), lateral head of the triceps (TRI) and the diaphragm (DIA) ( Figure 1). Muscle samples were collected into cryovials and snap-frozen in liquid nitrogen, before transfer to -80°C storage until use.

### MicroRNA extraction

Frozen muscle samples were crushed with a pestle and mortar that was pre-chilled on dry ice. Tissue powder was collected into a 1.5ml Eppendorf. MicroRNA was extracted using a Qiagen miRNeasy kit (Qiagen, #217084), according to manufacturer’s instructions. RNA concentration and purity were analysed using a NanoDrop 1000 spectrophotometer (ThermoFisher Scientific). Samples with a 260 nm:230 nm absorbance ratio of less than 1.7 underwent a further isopropanol precipitation step. Samples were stored at -20°C.

### Reverse transcription

MicroRNA was reverse transcribed using a miRCURY LNA RT kit (Qiagen, #339340) with the addition of the provided UniSp6 RNA spike in control and the cel-miR-39-3p RNA spike in template (Qiagen, #339390), according to manufacturer’s instructions. Samples were diluted to a concentration of 5ng/μl in nuclease free H _2_O and were reverse transcribed using 10ng total RNA per 10μl reaction.

### miFINDER Panel qPCR

cDNA from vastus lateralis samples of 4 WT (WT-P3, -Q5, -T3 and -Y3) and 4 DE50-MD (DE50-H6, -P1, -T6 and -Y1) 6-month-old dogs underwent quantitative PCR using the Dog miFinder Focus V2, miRCURY LNA miRNA Focus PCR panel (Qiagen, #339325, YAFD-201YG-2). The plates were in 384-well format, with wells pre-filled with primers to a panel of 87 miRs of interest, RNA spike-ins UniSp6 and cel-miR-39-3p (four replicate pre-filled wells of each target per plate), and the interplate calibrator UniSp3 IPC (eight replicate pre-filled wells per plate). The interplate calibrator wells contained primers and a DNA template for UniSp3 IPC. qPCR was conducted on a CFX384 light-cycler (BioRad) with the following cycling parameters: heat activation 95°C 2 minutes; 2-step cycling (95°C for 10 seconds and 56°C for 60 seconds) for 40 cycles; melt-curve analysis 60–95°C. Samples were tested across 2 miFinder Panel plates (2 WT and 2 DE50-MD samples per plate) and results were normalised to the interplate calibrator (see Underlying data). Given the breadth of this initial RNA panel, only a single well was used per target, per sample.

### LNA miRCURY assay qPCR

**Figure 2.**
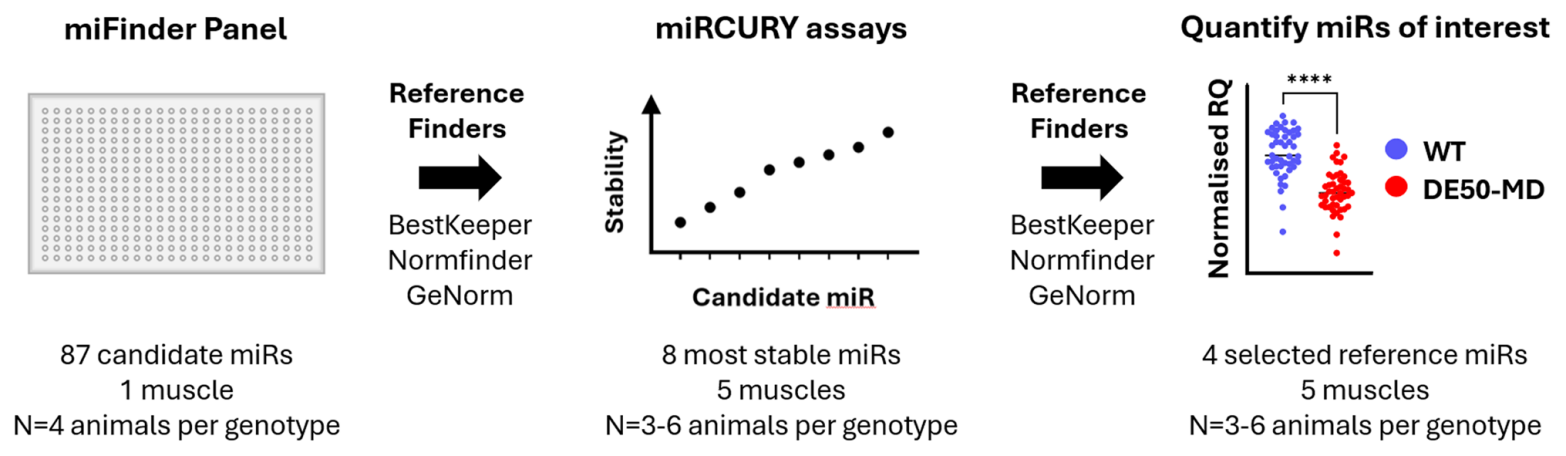
Workflow for identification of reference microRNAs. The number of microRNAs (miRs), number of muscles and number of study animals per genotype used for each stage of the methods.

Based on initial results from the miFinder panel, cDNA was prepared (as above) from VL muscle biopsy samples collected longitudinally from 6 WT and 6 DE50-MD dogs at 3, 6, 9, 12, 15 and 18-months of age, and from CRT, DIA, TRI and SEM muscles collected post-mortem from 3 WT and 3 DE50-MD dogs. Sample sizes were selected based on sample size calculations for other biomarkers of the DE50-MD phenotype that were determined for this same cohort of dogs ( Riddell *et al.*, 2021; Riddell *et al.*, 2022). Muscle samples were unavailable for 1 DE50-MD 15-month VL and 1 WT 18-month VL, thus our complete dataset consisted of 70 longitudinal VL biopsy samples and 24 post-mortem muscle samples ( Figure 1). cDNA was used in qPCR assays with primers (Qiagen, #339306) to a select panel of potential reference RNAs: cfa-miR-15a (#YP02103582), hsa-let-7b-5p (#YP00204750), hsa-miR-29b-3p (#YP00204679), U6 snRNA (v2) (#YP02119464), hsa-miR-106b-5p (#YP00205884), hsa-miR-130a-3p (#YP00204658), cfa-miR-191 (#YP00205972), cfa-miR-125a (#YP00203952), hsa-miR-27b-3p (#YP00205915), spike-in controls cel-miR-39-3p (#YP00203952) and UniSp6 (#YP00203954), and interplate calibrator hsa-miR-128-3p (#YP00205995). qPCR was performed in duplicate using the miRCURY LNA SYBR Green PCR Kit (Qiagen, #339347), according to manufacturer’s instructions (PCR cycling parameters as above for the miFinder Panel qPCR assay). Interplate calibration was achieved by using a single plate to quantify a subset of miRs in samples from each original plate (N=4 common miRCURY assays per plate); the mean difference in Cq value was determined for each plate and was subtracted from the original Cq result to give the adjusted mean Cq (see Underlying data). Following establishment of a panel of reference miRs, qPCR assays were performed using primers to the following group of miRs of interest ( Figure 2): cfa-miR-1 (#YP02119135), hsa-miR-133a-3p (#YP00204788), hsa-miR-206 (#YP00206073) and hsa-miR-214-3p (#YP00204510).

### Reference finders

geNorm, Bestkeeper (modified by the authors for miFinder panel analysis to accommodate 73 reference candidates and up to 94 samples, see Extended data ( Riddell *et al.*, 2024b)) and Normfinder reference gene finder algorithms were used initially on the miFinder panel data for the full dataset (N=4 for each genotype) and also for each genotype individually. Input data for each algorithm were as follows: geNorm used raw Cq values that were converted to relative quantities (RQs) within the software; Bestkeeper used raw Cq values; Normfinder used log relative quantities. For individual LNA miRCURY qPCR assays geNorm (Qbase+), Bestkeeper and Normfinder analyses were performed on the full dataset and also on the following subsets of the dataset:

• All samples (N=94)
  ◦ All WT samples (N=47)
  ◦ All DE50-MD samples (N=47)
• Vastus Lateralis samples only (N=70)
  ◦ WT vastus lateralis samples only (N=35)
  ◦ DE50-MD vastus lateralis samples only (N=35)

• Post-mortem (PM) 18-month samples only (N=36)
  ◦ DE50-MD PM samples only (N=18)
  ◦ WT PM samples only (N=18)

For grouped Normfinder analysis, grouping criteria were as follows:

• Individual animal (N=17)
• WT/DE50-MD (2 groups)
• Muscle type (5 groups: VL, CRT, SEM, TRI, DIA)
• Age (6 groups)

### Statistical analysis

Unpaired, two-tailed T-tests were used to compare results between WT and DE50-MD genotypes (all samples) for log-RQ of potential reference RNAs and miRs of interest miR-1, -133a, -206 and -214, corrected for multiple comparisons by the Holm–Šídák method. Linear mixed models were used to compare results between different muscle groups, between WT and DE50-MD genotypes, and between muscle groups within each genotype for log-RQ of potential reference RNAs and miRs of interest miR-1, -133a, -206 and -214, with *post hoc* analysis by Holm–Šídák correction for multiple comparisons. For all statistical tests, P values of less than 0.05 were considered statistically significant. *Post-hoc* comparisons of age-associated effects were only performed if age was determined to be statistically significant within the linear mixed model. T-tests were performed using GraphPad Prism Version 9.0.0 and linear mixed models were performed using IBM SPSS Statistics Version 29; free software alternatives that would be suitable include R or JASP.

## Results

### miFINDER panel

cDNA from N=4 WT and N=4 DE50-MD 6-month-old VL samples was assessed using the Dog miFinder Focus V2, miRCURY LNA miRNA Focus PCR panel. Of the 87 candidate RNAs, 14 had high Cq values (Cq>32 in all 8 samples and with undetectable quantities in at least 1 sample), suggesting very low levels or absence of these transcripts (miR-122, -124, -137, -141, -182, -183, -184, -200a, -200b, -203, -205, -34c, -375, -96): these candidates were thus omitted from further analyses. GeNorm, Bestkeeper and Normfinder reference gene finder analysis was thus carried out on a total of 8 samples, with 73 candidate reference genes. Results were analysed as a group (All), WT dogs alone (WT) or DE50-MD dogs alone (DE50-MD).

### geNorm M values

**Figure 3.**
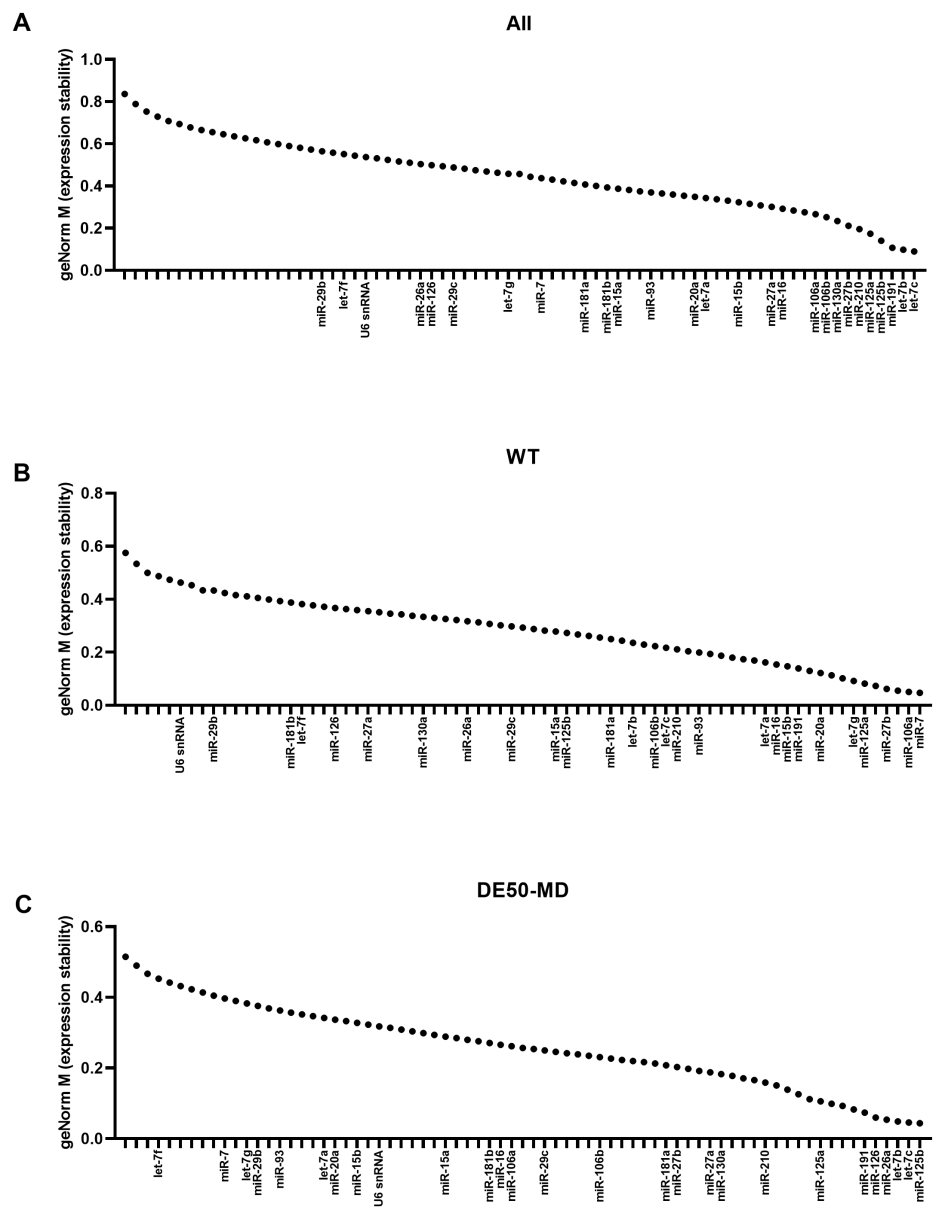
geNorm M expression stability values for miFinder panel. Results from least stable (high M values) to most stable (low M values) for RNA expression in vastus lateralis muscle from 6-month-old dogs (WT: N=4; DE50-MD: N=4) for **A**) all samples grouped together, **B**) WT samples only and **C**) DE50-MD samples only. X-axis labels are redacted to show only key miRs of interest to the study.

Overall, the large miR panel displayed a smooth continuum of stability values, with many candidates achieving the commonly-accepted M<0.5 threshold for suitability ( Figure 3A). Assessment of WT ( Figure 3B) or DE50-MD samples alone ( Figure 3C) lowered M values globally, implying considerable disease-associated differences. Confirming this, miR-7 and miR-106a were the highest ranking candidates in WT samples, yet both scored poorly in DE50-MD, while miR-26a and miR-126 were high ranking in DE50-MD, but poor scoring in WT. Members of the let-7 family let-7c and let-7b scored highly for the dataset as a whole and for the DE50-MD genotype alone, while let-7g also ranked in the top 10 most stable genes for WT alone ( Figure 3 and Table 1). miR-191 and miR-125a also performed well across all 3 groups. In contrast, the commonly employed reference RNA snRNA U6 scored comparatively poorly in all comparisons, coming 51 ^st^ out of 73 candidates for stability across all samples (see Extended data Table S1 ( Riddell *et al.*, 2024b)). 

**Table 1.** Top 20 potential reference RNAs by Qbase+ geNorm analysis. geNorm results for the miFinder panel of RNAs in vastus lateralis muscle from 6-month-old dogs (WT: N=4; DE50-MD: N=4). Lists of the top 20 most stable RNAs from most stable (lowest geNorm M expression stability values) to least stable for all samples together, WT samples alone and DE50-MD samples alone. RNAs that were common across all 3 groupings are highlighted in bold.

	All	WT	DE50-MD
Most stable	let-7c	miR-7	miR-125b
	let-7b	miR-106a	let-7c
	**miR-191**	miR-195	let-7b
	miR-125b	miR-27b	miR-26a
	**miR-125a**	miR-23a	miR-126
	miR-210	**miR-125a**	**miR-191**
	miR-27b	let-7g	miR-30d
	miR-130a	**miR-335**	miR-195
	miR-106b	miR-24	miR-21
	miR-106a	miR-20a	**miR-125a**
	**RNU1A1**	**RNU1A1**	miR-143
	miR-196a	**miR-191**	miR-214
	miR-16	miR-15b	**RNU1A1**
	miR-27a	miR-16	miR-34b
	**miR-335**	let-7a	miR-210
	miR-92a	miR-30c	**miR-335**
	miR-15b	miR-1	miR-148a
	miR-24	miR-145	miR-103
	miR-23a	miR-92a	miR-130a
Least stable	let-7a	miR-499	miR-27a

### Bestkeeper Pearson correlation coefficient

**Figure 4.**
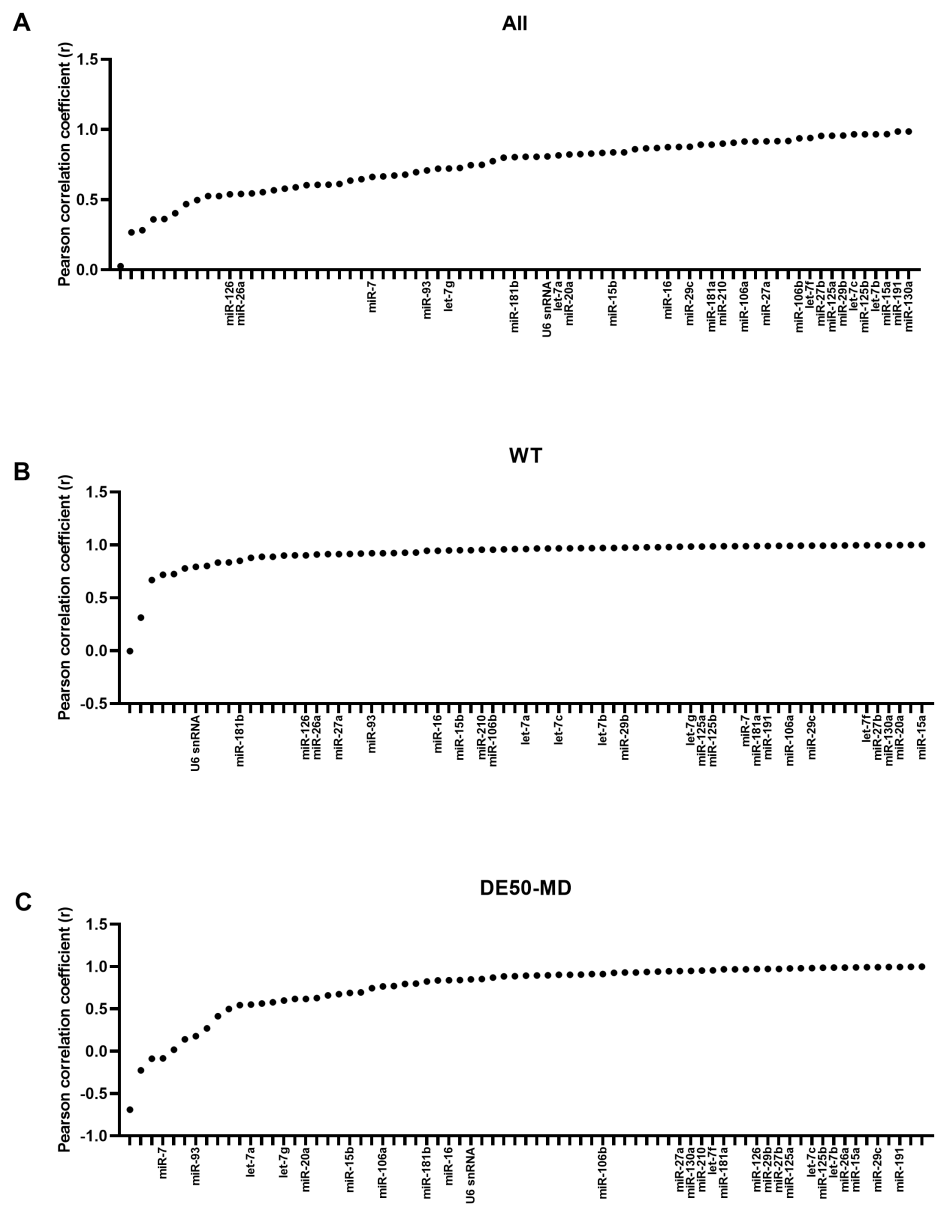
Bestkeeper Pearson correlation coefficients for miFinder panel. Results from least stable (low r values) to most stable (high r values) for RNA expression in vastus lateralis muscle from 6-month-old dogs (WT: N=4; DE50-MD: N=4) for **A**) all samples grouped together, **B**) WT samples only and **C**) DE50-MD samples only. X-axis labels are redacted to show only key miRs of interest to the study.

As with geNorm analysis, Bestkeeper showed markedly greater stability in genotype-restricted comparisons than for the entire dataset: for both WT and DE50-MD, correlation with the consensus was greater than 0.8 for the bulk of the panel, with an abrupt decline in correlation only in the lowest ranking 5–10 candidates. Accordingly, candidate rankings also differed between comparisons. let-7b and let-7c were ranked highly overall and in DE50-MD muscle, but less so in WT muscle alone; indeed, miR-191, miR-125b, miR-15a, miR-27b, let-7f and miR-181a were the only miRs that scored in the top 20 most stable RNAs across all 3 Bestkeeper groups ( Figure 4 and Table 2). snRNA U6 performed relatively poorly for stability, being ranked 34 ^th^, 67 ^th^ and 42 ^nd^ for the whole dataset, WT only and DE50-MD only groups, respectively (see Extended data Table S2 ( Riddell *et al.*, 2024b)).

**Table 2.** Top 20 potential reference RNAs by Bestkeeper analysis. Bestkeeper results for the miFinder panel of RNAs in vastus lateralis muscle from 6-month-old dogs (WT: N=4; DE50-MD: N=4). Lists of the top 20 most stable RNAs from most stable (highest Bestkeeper Pearson correlation coefficients (r)) to least stable for all samples together, WT samples alone and DE50-MD samples alone. RNAs that were common across all 3 groupings are highlighted in bold.

	All	WT	DE50-MD
Most stable	miR-130a	**miR-15a**	miR-17
	**miR-191**	miR-21	miR-22
	**miR-15a**	miR-20a	**miR-191**
	let-7b	miR-130a	miR-34a
	**miR-125b**	**miR-27b**	miR-29c
	let-7c	**let-7f**	miR-30b
	miR-29b	miR-34a	**miR-15a**
	miR-125a	miR-103	miR-26a
	**miR-27b**	miR-224	let-7b
	**let-7f**	miR-146b	**miR-125b**
	miR-106b	miR-29c	let-7c
	miR-92a	miR-195	miR-30d
	RNU1A1	miR-106a	miR-125a
	miR-27a	miR-24	**miR-27b**
	miR-196a	**miR-191**	miR-29b
	miR-106a	**miR-181a**	miR-126
	miR-148a	miR-7	miR-133b
	miR-210	miR-30c	5S rRNA
	**miR-181a**	miR-218	**miR-181a**
Least stable	5S rRNA	**miR-125b**	**let-7f**

### Normfinder stability values

**Figure 5.**
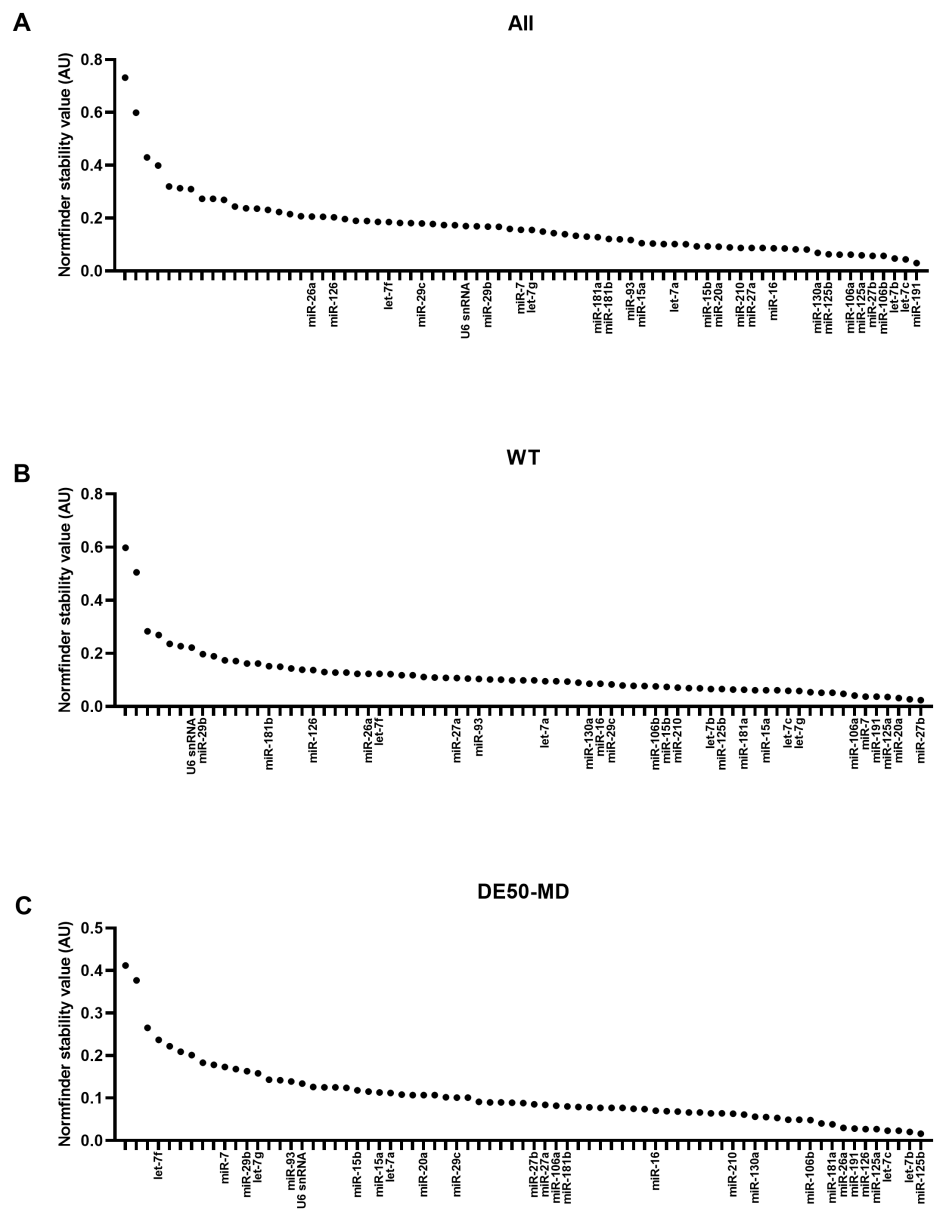
Normfinder values for miFinder panel. Results from least (high Normfinder stability value arbitrary units (AU)) to most stable (low AU) for RNA expression in vastus lateralis muscle from 6-month-old dogs (WT: N=4; DE50-MD: N=4) for **A**) all samples grouped together, **B**) WT samples only and **C**) DE50-MD samples only. X-axis labels are redacted to show only key miRs of interest to the study.

In agreement with geNorm and Bestkeeper analyses, miR-191 and miR-125a scored highly across all 3 groups of data ( Figure 5 and Table 3). Let-7b and let-7c were also in the top 20 most stable RNAs for all groups. snRNA U6 again scored relatively poorly, coming 42 ^nd^, 67 ^th^ and 57 ^th^ most stable out of 73 candidates for all data, WT only and DE50-MD only respectively (see Extended data Table S3 ( Riddell *et al.*, 2024b)).

**Table 3.** Top 20 potential reference RNAs by Normfinder analysis (ungrouped). Normfinder results for the miFinder panel of RNAs in vastus lateralis muscle from 6-month-old dogs (WT: N=4; DE50-MD: N=4). Lists of the top 20 most stable RNAs from most stable (lowest Normfinder stability values (AU)) to least stable for all samples together, WT samples alone and DE50-MD samples alone. RNAs that were common across all 3 groupings are highlighted in bold.

	All	WT	DE50-MD
Most stable	**miR-191**	miR-27b	**miR-125b**
	**let-7c**	miR-195	**let-7b**
	**let-7b**	miR-20a	miR-30d
	miR-106b	**miR-125a**	**let-7c**
	miR-27b	**miR-191**	**miR-125a**
	**miR-125a**	miR-7	miR-126
	miR-106a	miR-106a	**miR-191**
	RNU1A1	miR-23a	miR-26a
	**miR-125b**	miR-23b	miR-181a
	miR-130a	RNU1A1	miR-195
	miR-24	miR-224	miR-106b
	miR-335	let-7g	miR-133a
	miR-16	**let-7c**	miR-30b
	miR-92a	miR-451	miR-21
	miR-27a	miR-15a	miR-101
	miR-23a	miR-24	miR-130a
	miR-210	miR-181a	miR-143
	miR-196a	miR-335	miR-210
	miR-20a	**miR-125b**	miR-103
Least stable	miR-15b	**let-7b**	miR-133b

### miFINDER panel conclusions

Our miFinder results (many candidates, few samples) were used to identify a shortlist of 8 potential reference miRs, based on consistent high rankings under all 3 reference finder approaches used. miR-130a, miR-191, miR-125a, let-7b, miR-15a, miR-27b, miR-29b and miR-106b all scored in the top 11 most stable RNAs for all 3 reference finder algorithms (with the exception of miR-15a and miR-29b, which scored very highly in Bestkeeper analyses, but less well under geNorm or Normfinder). snRNA U6 was also included in our further investigations to better place this work in context: although this small RNA scored poorly compared to other candidates in the miFinder panel, it is the most commonly used reference RNA used for miR normalisation in the literature.

### Individual qPCR assays

To identify the most suitable references from our shortlist, we used each candidate in individual qPCR assays, with a broader muscle sample collection. For this work, we used cDNA from longitudinal (3, 6, 9, 12, 15 and 18 month) VL muscle biopsy samples (N=6 dogs per age group, per genotype), and 18-month post-mortem muscles from N=3 dogs per genotype: CRT, SEM, TRI and DIA. VL samples were unavailable for analysis for 1 WT dog aged 18 months and 1 DE50-MD dog aged 15 months (see Methods Figure 1). Data were assessed as above (whole dataset, WT/DE50-MD subsets), and where appropriate, also analysed by muscle (VL only) or by age (18-month samples only), again either as a whole, or as WT/DE50-MD restricted subsets.

### GeNorm M values

**Figure 6.**
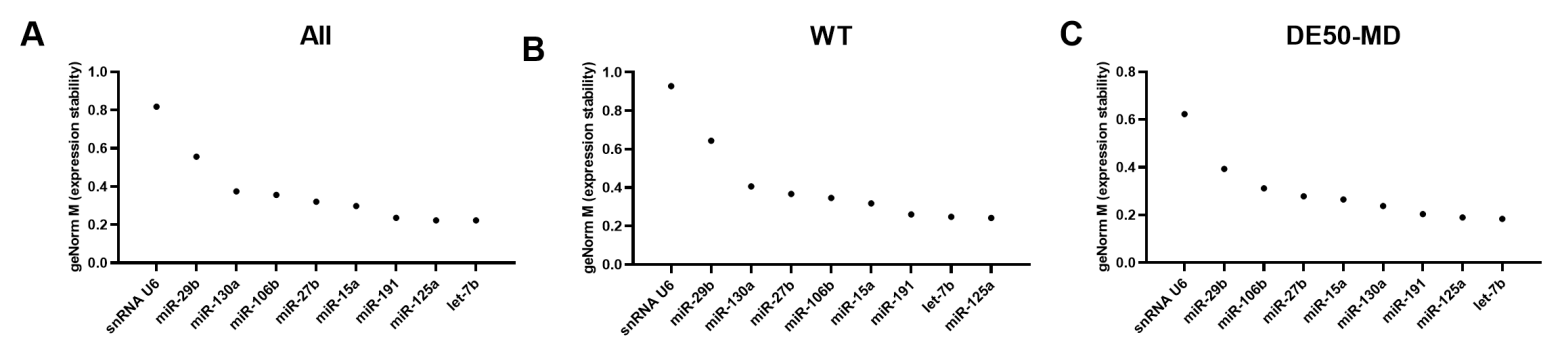
geNorm M-values. Results from least stable (high M values) to most stable (low M values) for RNA expression in muscle samples from WT and DE50-MD dogs aged 3 to 18 months (VL: N=6 per genotype, per timepoint (3-, 6-, 9-, 12-, 15- and 18-months); cranial tibial, lateral triceps, semimembranosus and diaphragm: N=3 per genotype, 18-months of age)) for **A**) all samples grouped together, **B**) WT samples only and **C**) DE50-MD samples only.

GeNorm analysis with our wider muscle sample collection revealed substantially greater agreement across subsets: assessment of the dataset as a whole or restricted by genotype, VL muscle only or 18-month samples only, revealed that miR-125, let-7b and miR-191 consistently scored highly, while miR-29b and snRNA-U6 were the least stable candidates in all cases, and indeed were often the only candidates with M>0.5 ( Figure 6 and Table 4).

**Table 4.** GeNorm results for panel of nine potential reference RNAs. List of geNorm results from most stable (high M values) to least stable (low M values) for all samples together (N=94), WT samples (N=47), DE50-MD samples (N=47), all vastus lateralis (VL) samples (N=70), WT VL samples (N=35), DE50-MD VL samples (N=35), samples from 18-month-old dogs (N=35), WT 18-months samples (N=17), DE50-MD 18-months samples (N=18).

	All	WT	DE50-MD	VL	WT VL	DE50-MD VL	18m	WT 18m	DE50-MD 18m
Most stable	miR-125a	miR-125a	let-7b	let-7b	miR-125a	let-7b	miR-125a	miR-125a	let-7b
	let-7b	let-7b	miR-125a	miR-125a	let-7b	miR-125a	let-7b	let-7b	miR-125a
	miR-191	miR-191	miR-191	miR-191	miR-191	miR-191	miR-191	miR-191	miR-106b
	miR-15a	miR-15a	miR-130a	miR-27b	miR-15a	miR-130a	miR-106b	miR-27b	miR-191
	miR-27b	miR-106b	miR-15a	miR-15a	miR-27b	miR-27b	miR-130a	miR-130a	miR-15a
	miR-106b	miR-27b	miR-27b	miR-130a	miR-106b	miR-15a	miR-15a	miR-106b	miR-130a
	miR-130a	miR-130a	miR-106b	miR-106b	miR-130a	miR-106b	miR-27b	miR-15a	miR-27b
	miR-29b	miR-29b	miR-29b	miR-29b	miR-29b	miR-29b	miR-29b	miR-29b	miR-29b
Least stable	snRNA U6	snRNA U6	snRNA U6	snRNA U6	snRNA U6	snRNA U6	snRNA U6	snRNA U6	snRNA U6

### Bestkeeper Pearson correlation coefficient

**Figure 7.**
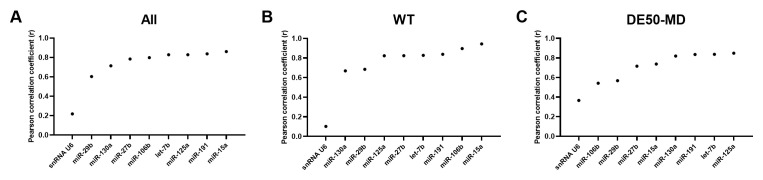
Bestkeeper Pearson Correlation Coefficient. Results from least stable (low r values) to most stable (high r values) for RNA expression in muscle samples from WT and DE50-MD dogs aged 3 to 18 months (vastus lateralis: N=6 per genotype, per timepoint (3-, 6-, 9-, 12-, 15- and 18-months); cranial tibial, lateral triceps, semimembranosus and diaphragm: N=3 per genotype, 18-months of age)) for **A**) all samples grouped together, **B**) WT samples only and **C**) DE50-MD samples only.

Under Bestkeeper analysis, miR-29b and snRNA U6 ranked poorly for all groupings analysed (in agreement with geNorm), with the exception of miR-29b in WT 18-month-old samples ( Figure 7 and Table 5). For high-scoring candidates, miR-15a consistently performed well (Pearson correlation coefficient r value of 0.74 or higher across all groupings) although it typically ranked fractionally higher for WT than DE50-MD groups. Let-7b, miR-125a and miR-191 also performed well with minimum r values of 0.82, 0.76 and 0.75 respectively when comparing all groupings.

**Table 5.** Bestkeeper Pearson Correlation Coefficient. List of Bestkeeper results from most stable (low r values) to least stable (high r values) for all samples together (N=94), WT samples (N=47), DE50-MD samples (N=47), all vastus lateralis (VL) samples (N=70), WT VL samples (N=35), DE50-MD VL samples (N=35), samples from 18-month-old dogs (N=35), WT 18-months samples (N=17), DE50-MD 18-months samples (N=18).

	All	WT	DE50-MD	VL	WT VL	DE50-MD VL	18m	WT 18m	DE50-MD 18m
Most stable	miR-15a	miR-15a	miR-125a	miR-125a	miR-15a	miR-15a	miR-15a	miR-106b	miR-125a
	miR-191	miR-106b	let-7b	miR-191	miR-27b	miR-191	miR-106b	miR-15a	let-7b
	miR-125a	miR-191	miR-191	let-7b	miR-191	miR-125a	miR-130a	miR-130a	miR-191
	let-7b	let-7b	miR-130a	miR-27b	miR-125a	let-7b	let-7b	miR-29b	miR-130a
	miR-106b	miR-27b	miR-15a	miR-15a	let-7b	miR-106b	miR-191	let-7b	miR-27b
	miR-27b	miR-125a	miR-27b	miR-106b	miR-106b	miR-27b	miR-125a	miR-125a	miR-15a
	miR-130a	miR-29b	miR-29b	miR-130a	miR-130a	miR-130a	miR-27b	miR-191	miR-29b
	miR-29b	miR-130a	miR-106b	miR-29b	miR-29b	miR-29b	miR-29b	miR-27b	miR-106b
Least stable	snRNA U6	snRNA U6	snRNA U6	snRNA U6	snRNA U6	snRNA U6	snRNA U6	snRNA U6	snRNA U6

### Normfinder stability values

**Figure 8.**
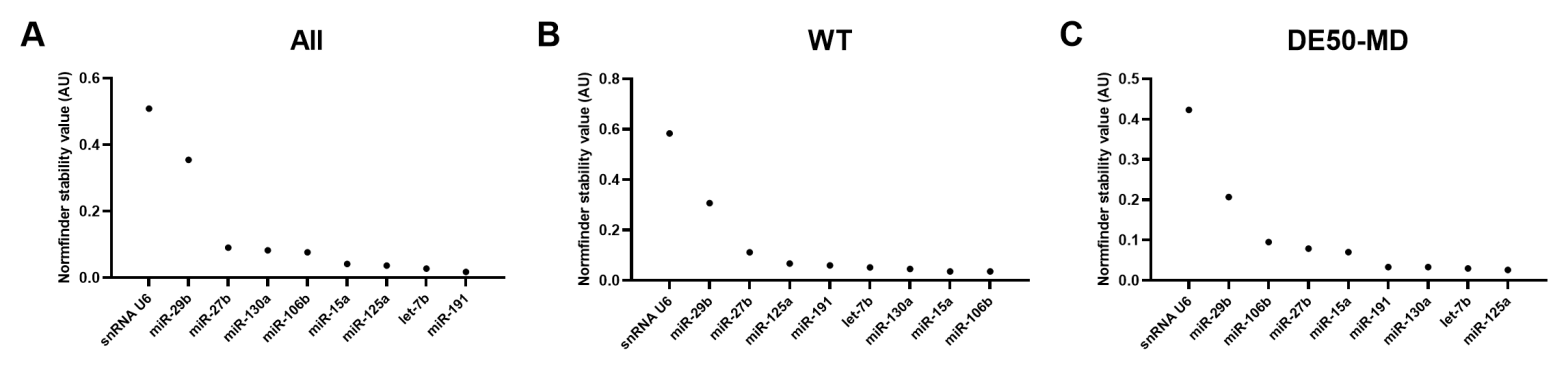
Normfinder stability values (ungrouped). Results from least stable (high stability value) to most stable (low stability value) for RNA expression in muscle samples from WT and DE50-MD dogs aged 3 to 18 months (vastus lateralis: N=6 per genotype, per timepoint (3-, 6-, 9-, 12-, 15- and 18-months); cranial tibial, lateral triceps, semimembranosus and diaphragm: N=3 per genotype, 18-months of age)) for **A**) all samples grouped together, **B**) WT samples only and **C**) DE50-MD samples only.

When analysing all data or each genotype individually (ungrouped), let 7b, miR-15a and miR-191 consistently ranked in the top 5 of the 9 genes analysed, all with Normfinder stability values of less than 0.07 AU, indicating very high stability ( Figure 8 and Table 6). In agreement with both geNorm and Bestkeeper, miR-29b and snRNA U6 were the least stable genes - and markedly so. Grouped analysis accounting for genotype, individual (dog ID), age and muscle type showed miR-191 and let-7b to be the most consistent in the rankings, scoring in the top 4 most stable genes across 10 groupings ( Table 7). miR-15 was the most stable for grouped analysis in WT dogs, whereas miR-125a and miR-130a were consistently in the top 4 most stable RNAs for grouped DE50-MD analysis.

**Table 6.** Normfinder stability values rankings ungrouped. List of ungrouped Normfinder results from most stable (high stability value arbitrary units (AU)) to least stable (low stability value AUs) for all samples together (N=94), WT samples (N=47), DE50-MD samples (N=47), all vastus lateralis (VL) samples (N=70), WT VL samples (N=35), DE50-MD VL samples (N=35), samples from 18-month-old dogs (N=35), WT 18-months samples (N=17), DE50-MD 18-months samples (N=18).

	All	WT	DE50-MD	VL	WT VL	DE50-MD VL	18m	WT 18m	DE50-MD 18m
Most Stable	miR-191	miR-15a	miR-125a	miR-191	miR-191	miR-130a	miR-15a	miR-106b	miR-125a
	let-7b	miR-106b	let-7b	miR-125a	miR-15a	miR-191	miR-106b	miR-15a	miR-191
	miR-125a	miR-130a	miR-130a	let-7b	miR-27b	miR-125a	miR-130a	miR-130a	let-7b
	miR-15a	let-7b	miR-191	miR-15a	miR-125a	let-7b	let-7b	let-7b	miR-130a
	miR-106b	miR-191	miR-15a	miR-27b	let-7b	miR-15a	miR-125a	miR-125a	miR-15a
	miR-130a	miR-125a	miR-27b	miR-130a	miR-106b	miR-27b	miR-191	miR-191	miR-106b
	miR-27b	miR-27b	miR-106b	miR-106b	miR-130a	miR-106b	miR-27b	miR-27b	miR-27b
	miR-29b	miR-29b	miR-29b	miR-29b	miR-29b	miR-29b	miR-29b	miR-29b	miR-29b
Least stable	snRNA U6	snRNA U6	snRNA U6	snRNA U6	snRNA U6	snRNA U6	snRNA U6	snRNA U6	snRNA U6

**Table 7.** Normfinder stability values rankings grouped. List of Normfinder results from most stable (high stability value arbitrary units (AU)) to least stable (low stability value AUs) for all samples together (N=94) grouped by genotype, dog ID, age or muscle, and for WT samples (N=47) and DE50-MD samples (N=47) grouped by dog ID, age or muscle.

	All				WT			DE50-MD		
	Genotype	Dog ID	Age	Muscle	Dog ID	Age	Muscle	Dog ID	Age	Muscle
Most stable	miR-191	miR-191	miR-191	miR-106b	miR-15a	miR-15a	miR-15a	miR-191	miR-191	let-7b
	let-7b	let-7b	let-7b	let-7b	miR-106b	miR-191	miR-106b	miR-125a	miR-130a	miR-191
	miR-125a	miR-125a	miR-125a	miR-191	miR-191	miR-125a	let-7b	let-7b	let-7b	miR-125a
	miR-130a	miR-15a	miR-15a	miR-125a	let-7b	let-7b	miR-191	miR-130a	miR-125a	miR-106b
	miR-27b	miR-106b	miR-130a	miR-15a	miR-125a	miR-106b	miR-125a	miR-15a	miR-15a	miR-130a
	miR-15a	miR-130a	miR-27b	miR-130a	miR-27b	miR-27b	miR-130a	miR-106b	miR-27b	miR-15a
	miR-106b	miR-27b	miR-106b	miR-27b	miR-130a	miR-130a	miR-27b	miR-27b	miR-106b	miR-27b
	miR-29b	miR-29b	miR-29b	miR-29b	miR-29b	miR-29b	miR-29b	miR-29b	miR-29b	miR-29b
Least stable	snRNA U6	snRNA U6	snRNA U6	snRNA U6	snRNA U6	snRNA U6	snRNA U6	snRNA U6	snRNA U6	snRNA U6

### Individual assay conclusion

Of the 9 candidates tested, miR-191 and let-7b scored very highly across all 3 algorithms used, essentially regardless of muscle, age or genotype. miR-125a and miR-15a also consistently scored highly (and often higher than miR-191 or let-7b), but were slightly more variable between genotype-specific datasets. Assessed collectively, our results suggest that these 4 candidates, miR-191, let-7b, miR-125a and miR15a are highly suitable for normalising miR expression in WT and DE50-MD canine skeletal muscle.

### Candidate validation

**Figure 9.**
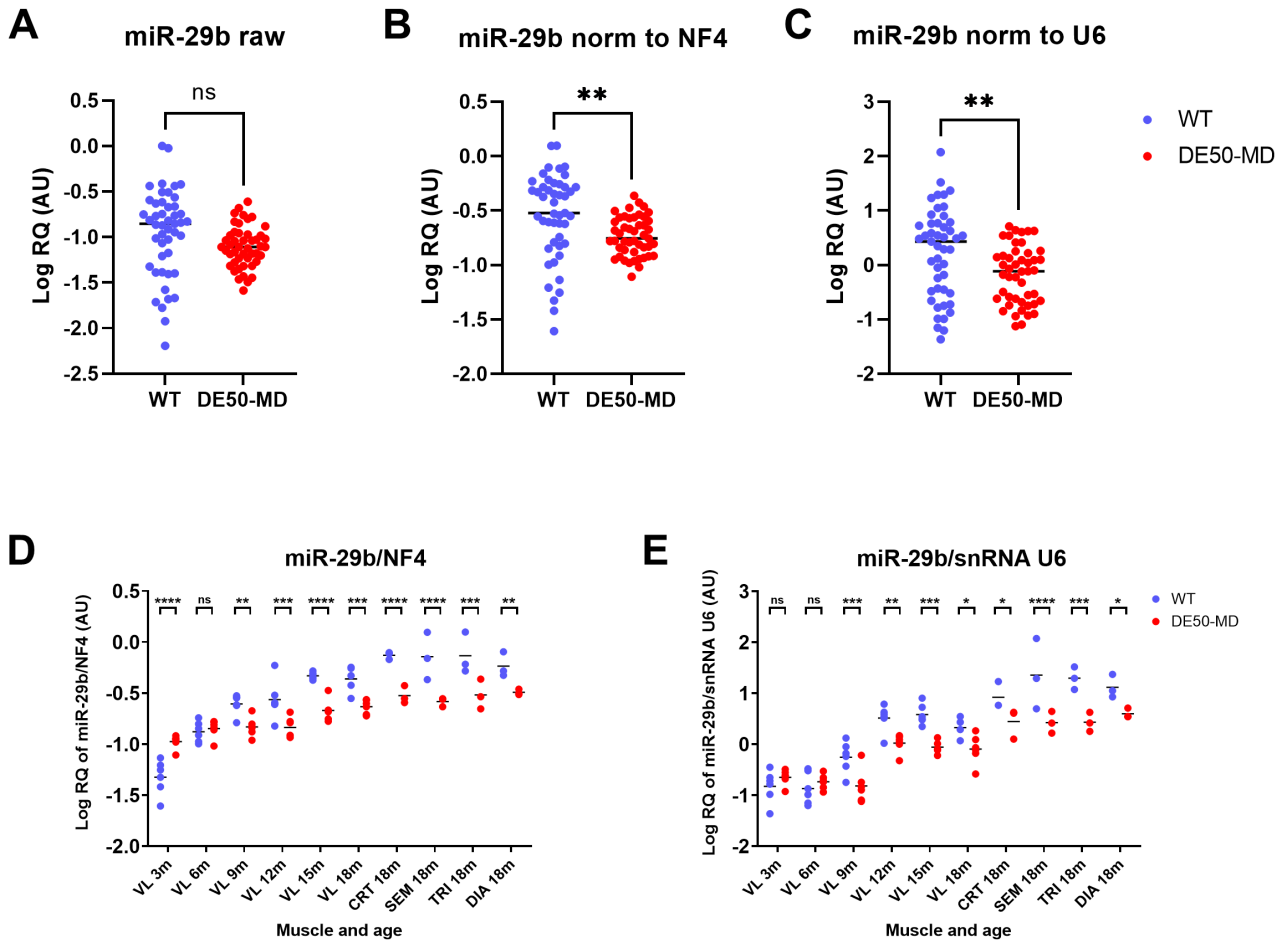
Validation of high scoring reference RNA candidates. WT (blue; N=47) and DE50-MD (red; N=47) dog muscle (N=6 vastus lateralis (VL) muscle samples longitudinally from dogs aged 3-,6-, 9-, 12-, 15- and 18-months; N=3 cranial tibial (CRT), semimembranosus (SEM), triceps (TRI) and diaphragm (DIA) muscle samples from 18-month-old dogs) log relative quantity (RQ) of miR-29b expression **A**) raw (not normalised) **B**) normalised to the geometric mean of the 4 best scoring potential reference miRs (NF4; geomean of RQ of miR-191, let-7b, miR125a and miR-15a) or **C**) normalised to snRNA U6. Results grouped by genotype, muscle and age for **D**) NF4-normalised miR-29b and **E**) snRNA U6-normalised miR-29b. Asterisks indicate the statistical significance of the difference between the WT and DE50-MD genotypes based on linear mixed model analysis: ns = not significant; *P<0.05; **P<0.01; ***P<0.001; ****P<0.0001.

The validity of the 4 highest performing reference miR candidates (miR-191, let-7b, miR-125a and miR-15a) was assessed using a within-dataset approach reported previously ( Hildyard *et al.*, 2019), using the strongest performing candidates to normalise the weakest. Accordingly, the geometric mean of the relative quantity (RQ) of the 4 best scoring RNAs (henceforth referred to as normalisation factor-4 (NF4)) was used to normalise the consistently low-scoring miR-29b. As snRNA U6 is a commonly used reference gene (although poor scoring in this study) we also used it to normalise miR-29b expression for comparison. Raw (before normalisation) expression of miR-29b did not significantly differ between WT and DE50-MD samples ( Figure 9 A). When normalised to either NF4 ( Figure 9 B) or to snRNA U6 ( Figure 9 C), miR-29b expression was significantly lower in DE50-MD compared to WT samples. Segregating these results by genotype, muscle group and age revealed a clear age-associated increase in miR-29b expression in both genotypes, but here use of NF4 normalisation allowed yet further nuance to be assessed. It showed that miR-29b expression in young (3-month) DE50-MD muscle was greater than in healthy muscle, but subsequent increases with age were more modest in dystrophic tissue, progressively, then falling below those in WT muscle samples. These distinctions were not apparent when U6 was used as a reference ( Figure 9 D and E).

**Figure 10.**
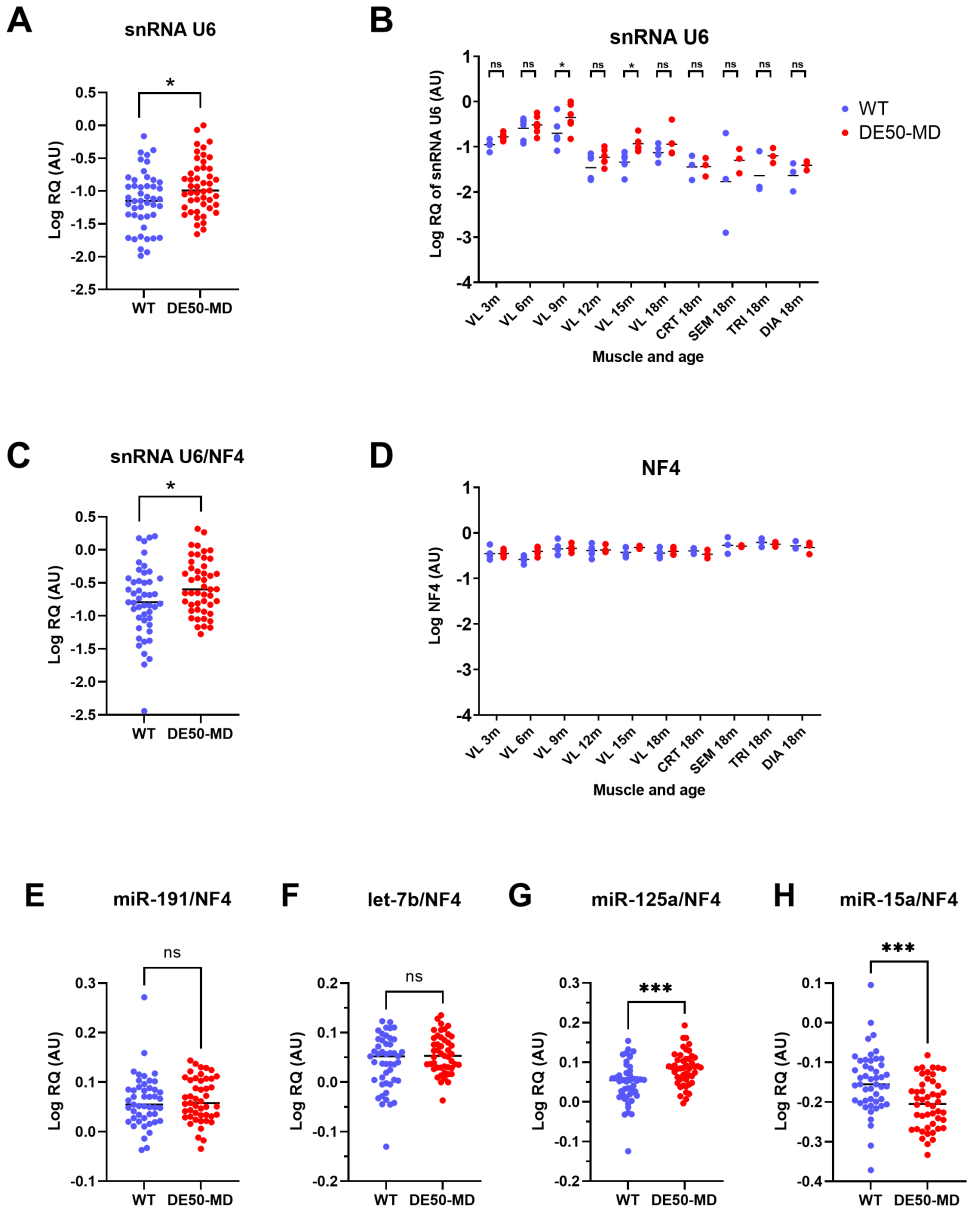
snRNA U6 expression. WT (blue; N=47) and DE50-MD (red; N=47) dog muscle (N=6 vastus lateralis (VL) muscle samples longitudinally from dogs aged 3-,6-, 9-, 12-, 15- and 18-months; N=3 cranial tibial (CRT), semimembranosus (SEM), triceps (TRI) and diaphragm (DIA) muscle samples from 18-month-old dogs) log relative quantity (RQ) of snRNA U6 expression **A**) grouped by genotype, **B**) grouped by genotype, muscle and age and **C**) normalised to NF4. **D**) Log geometric mean of the 4 best scoring potential reference miRs (NF4; geomean of RQ of miR-191, let-7b, miR-125a and miR-15a). **E**) Log relative quantity (RQ) of the individual reference RNA expressions normalised to the geometric mean of miR-191, let-7b, miR-125a and miR-15a (NF4) for **E**) miR-191, **F**) let-7b **G**) miR-125a and **H**) miR-15a. Asterisks indicate the statistical significance of the difference between the WT and DE50-MD genotypes based on linear mixed model analysis: ns = not significant; *P<0.05, ***P<0.001.

Raw (non-normalised) snRNA U6 RQ was modestly, but nevertheless, significantly higher in DE50-MD compared to WT muscle samples (1.8-fold increase, P=0.024; Figure 10 A). In post-hoc analyses, significant differences were detected between genotypes only at 9- and 15-months of age, however for both genotypes there was a significant decline with age in snRNA U6 expression (P<0.0001; Figure 10 B). Following normalisation of U6 to NF4, significant differences were retained both between genotypes (P=0.021) and muscle group (P<0.0001) ( Figure 10 C). Conversely, no such age-, genotype- or muscle-associated differences were observed in the geometric mean of the top 4 reference RNA candidates (NF4; Figure 10 D).

We next examined each of our high-scoring references (miR-191, let-7b, miR-125a and miR-15a) individually, following normalisation to the geometric mean of all four (NF4). Supporting their rankings via the three reference gene scoring approaches, there was no significant effect of genotype on miR-191 or let-7b. Conversely, miR-125a and miR-15a (which were more variably scored between genotypes) exhibited significant but modest increases (1.1-fold increase) and decreases (1.1-fold decrease), respectively, in DE50-MD compared to WT dogs ( Figure 10 E–H).

### Normalised expression of dystromiRs-1, -133a, -206 and fibromiR-214

**Figure 11.**
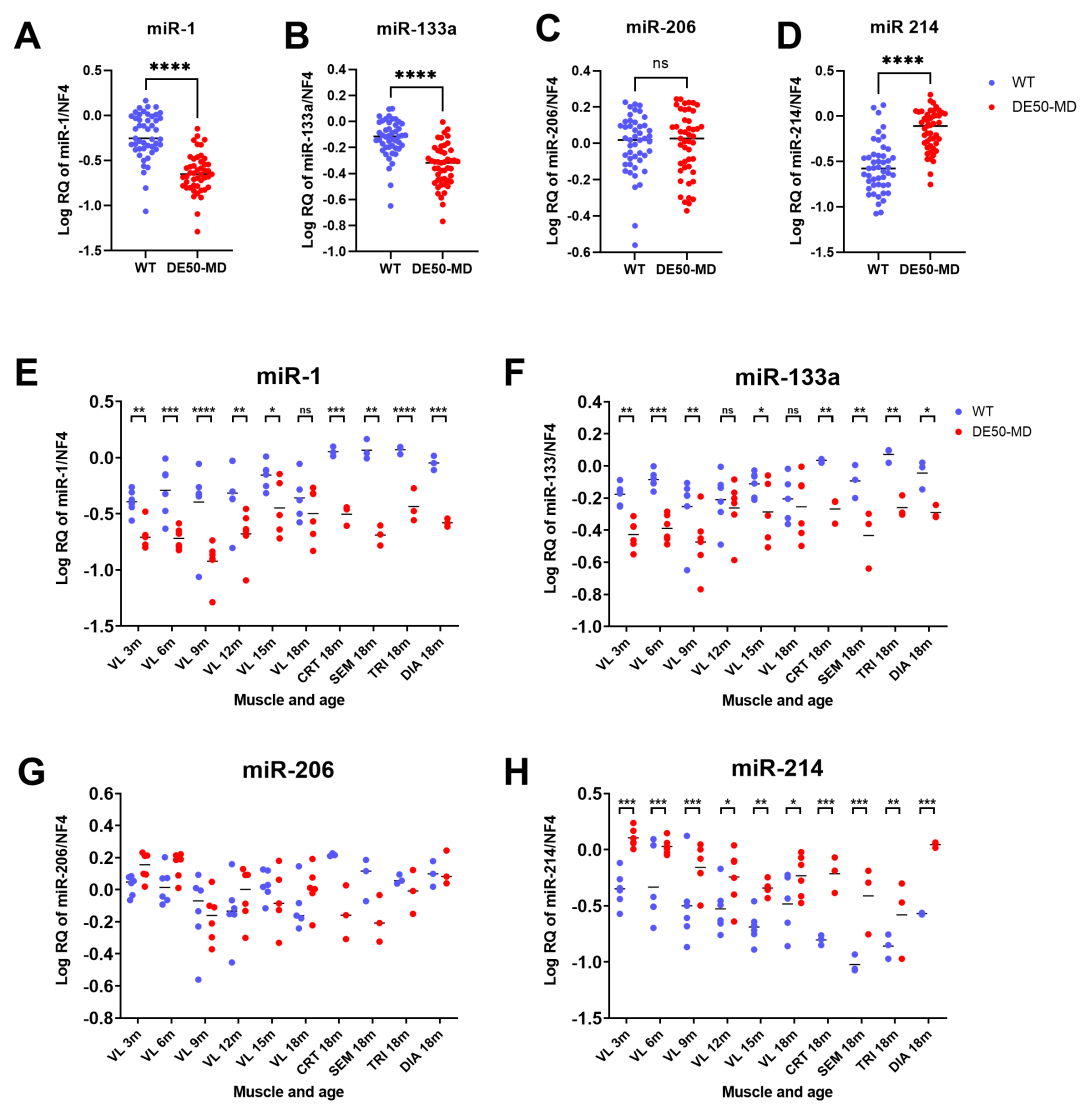
Expression of the dystromiRs miR-1, miR-133a and miR-206 and the fibromiR miR-214 normalised to the best performing reference genes (NF4). WT (blue; N=47) and DE50-MD (red; N=47) dog muscle (N=6 vastus lateralis (VL) muscle samples longitudinally from dogs aged 3-,6-, 9-, 12-, 15- and 18-months; N=3 cranial tibial (CRT), semimembranosus (SEM), triceps (TRI) and diaphragm (DIA) muscle samples from 18-month-old dogs) log relative quantity (RQ) of expression of miRs of interest normalised to the geometric mean of reference RNAs miR-191, miR-125a, let-7b and miR-15a (NF4) for dystromiRs **A**) miR-1, **B**) miR-133a, **C**) miR-206 and the fibromiR **D**) miR-214. Results grouped by genotype, muscle and age for **E**) miR-1/NF4, **F**) miR-133a/NF4, **G**) miR-206/NF4 and **H**) miR-214/NF4. Asterisks indicate the statistical significance of the difference between the WT and DE50-MD genotypes based on linear mixed model analysis: ns = not significant; *P<0.05; **P<0.01; ***P<0.001; ****P<0.0001.

Finally, to demonstrate the effectiveness of our reference candidates in a therapeutically relevant context, we measured expression of dystromiRs miR-1, miR-133a and miR-206, and fibromiR miR-214 in all test samples, using NF4 for normalisation. miR-1 and miR-133a were significantly lower in DE50-MD compared to WT muscle samples (miR-1: 3-fold decrease; miR-133a: 2-fold decrease; P<0.0001 for both, Figure 11 A–B), with post-hoc analysis showing these differences extended to all muscle groups (P<0.05) and almost all ages ( Figure 11 E–F). Conversely, no significant difference was found between genotypes for miR-206 expression (P=0.43, Figure 11 C and G). FibromiR miR-214 expression was significantly elevated in DE50-MD compared to WT muscle for every muscle group analysed (P<0.05, Figure 11 D and H). In 3- to 18-month VL samples, DE50-MD miR-214 expression was increased by a mean of 2-fold (+/- 0.4 SD), while in the 4 other muscle groups tested (18-month CRT, SEM, TRI and DIA) expression was increased by a mean of 4-fold (+/- 0.5 SD). 

## Discussion

Quantification of microRNAs (miRs) by RT-qPCR in the skeletal muscle of DMD patients or animal models of the disease can identify novel disease biomarkers and pathophysiological mechanisms. The use of normalisation factors for raw RT-qPCR data to control for unavoidable variation in RNA isolation and cDNA preparation is essential for accurate quantification of target miRs. miRs that are stably expressed between test samples can be used to normalise data from miRs of interest and allow comparisons between samples. The aim of this study was to identify and validate reference miR candidates for a range of skeletal muscle groups from the DE50-MD canine model of DMD and WT control dogs of different ages. Given the large numbers of potential candidate miRs, and the relative paucity of data on microRNA behaviour in the skeletal muscle of dystrophic dogs, we adopted a two-tiered approach: an initial primary screen (using many miRs and a modest number of samples) to identify strong candidates, followed by a more in-depth secondary analysis using those strong candidates in a large number of samples, including different ages and muscle types. Once identified, miRs of interest could be accurately quantified and compared.

Initial screening was performed using 6-month old VL samples from DE50-MD and WT controls. This age group was selected on the basis of phenotypic severity: while muscle damage is present at 3 months, we have previously shown that multiple biomarkers of the DE50-MD phenotype are nevertheless poorly resolved or not evident at this age, whereas 6-months of age sees the onset of reduced muscle mass ( Hornby *et al.*, 2021) and resistance to eccentric exercise compared to WT dogs ( Riddell *et al.*, 2023), alongside a peak in serum biomarkers of muscle damage ( Riddell *et al.*, 2021) and inflammation ( Riddell *et al.*, 2022). Quantification of 87 miRs in these samples produced a panel of potential reference miR/RNA candidates, based on stability scoring by the mathematical algorithms geNorm, BestKeeper and Normfinder. Each of the 3 algorithms assesses stability in different ways, with no one method being the gold standard ( Veryaskina *et al.*, 2022). Despite their differences in approach, the 3 reference finders shared many common RNAs in their top 20 ranked genes for stability across the whole dataset, with miR-130a, miR-191, miR-125a, miR-125b let-7b, let-7c, miR-27b and miR-106b universally scoring in the top 11. In addition, Bestkeeper scored miR-15a and miR-29b in the top 10 for stability, while geNorm ranked miR-210 in its top 10.

When healthy and dystrophic samples were analysed individually, the 3 programs also largely agreed, although geNorm and Normfinder shared more common stable RNAs than they did with BestKeeper. The top 20 most stable RNAs varied somewhat between the 2 genotypes, though several RNAs performed well in both groups. Of note, miR-191 uniquely scored in the top 20 genes of all three algorithms for the whole dataset, and for healthy or dystrophic subsets. miR-125a and miR-125b also scored in the top 20 for all groupings and algorithms, with the exception of WT samples only (Bestkeeper, miR-125a; geNorm, miR-125b). Members of the let-7 and miR-27 families of miRs scored highly for both genotypes in all algorithms, with let-7b, let-7c and miR-27b scoring as the most stable of their families overall. We also observed clear genotype-specific stable miRs: miR-30d scored well across all 3 reference finders in DE50-MD dogs but was not in the top 20 most stable genes for any WT-only analyses, while the reverse was true for miR-20a (disease-associated but otherwise stable expression suggests these miRs merit future investigation as therapeutic biomarkers).

Based on the miFinder panel results, miR-130a, miR-191, miR-125a, let-7b, miR-15a, miR-27b, miR-29b and miR-106b were shortlisted as potential reference miR candidates for the DE50-MD and WT study population. Despite mediocre performance in our initial screen, we also included snRNA U6 in our shortlist panel: this small RNA is a commonly used reference RNA for miR studies, and inclusion here thus serves to place this work in context.

Once a shortlisted panel was established, these potential reference miRs were assessed in our secondary screen, with a much larger collection of muscle samples. We quantified miR expression in VL muscle biopsies obtained longitudinally at 3-monthly intervals from 3–18 months of age, and also selected a panel of muscles that were collected post-mortem in dogs aged 18-months (CRT, SEM, TRI and DIA). The VL was chosen as this muscle is easily identified and superficially located, facilitating biopsy. This muscle is also commonly biopsied in human DMD patients (for the same reasons). Our post-mortem muscle panel, conversely, were selected to ensure representation of pelvic limb, thoracic limb and respiratory muscles. This collection of muscles allowed us to compare changes with increasing age (VL), and differences between muscles in 18-month old dogs, as well as comparing differences between genotypes: a potentially powerful dataset. 

Across the 3 reference gene assessment programs used, the majority of our panel exhibited very high stability: only snRNA U6 and miR-29b were clear exceptions, and often by a substantial margin. This was largely expected, given that all miRs were hand-picked from the top scoring candidates of our primary screen (with snRNA U6 included for comparative purposes). Despite this panel-wide strong performance, some candidates were demonstrably stronger than others: miR-191, miR-125a and let-7b consistently scored exceptionally highly for stability, regardless of genotype, age or muscle group. Interestingly, miR-191 has previously been proposed as a universal reference miR, due to its high stability across a range of normal human tissues ( Peltier & Latham, 2008). Subsequent studies have confirmed its suitability as a reference miR for colorectal adenocarcinoma patient serum ( Zheng *et al.*, 2013), breast cancer patient serum ( Hu *et al.*, 2012), Hepatitis B and hepatocellular carcinoma patient serum ( Li *et al.*, 2015), and bone marrow mesenchymal stromal cells *in vivo* under various conditions ( Costé & Rouleux-Bonnin, 2020). miR-191 regulates expression of genes involved in normal cell-cycling, apoptosis and cell proliferation ( Nagpal & Kulshreshtha, 2014), and several studies have shown its dysregulation in cancers including glioblastoma ( Xue *et al.*, 2019), ovarian tumours ( Jarych *et al.*, 2024; Takamizawa *et al.*, 2024), and pancreatic cancer ( Shi *et al.*, 2024). miR-125a is also abnormally expressed in several cancers, suggestive of a tumour-suppressor role in the regulation of cell proliferation, migration and apoptosis ( Fu & Cao, 2015; Guo *et al.*, 2009; Liang *et al.*, 2019). The *lethal-7* ( *let-7*) gene was the first miR identified in humans, and members of the let-7 family of miRs are highly conserved between species ( Pasquinelli *et al.*, 2000). Humans have 10 mature let-7 family isoforms, with key roles in termination of cell differentiation during development ( Roush & Slack, 2008). Our preliminary miR screening with the miFinder panel showed that multiple members of the let-7 family scored highly for stability; of these, let-7b scored highly most consistently across our groupings. Both let-7b and let-7c are associated with tumour suppressor roles in cancers, with lower expression associated with poor prognosis ( Nam *et al.*, 2008; Zhao *et al.*, 2014). In summary, miR-191, miR-125a and let-7b all have roles in normal cell-cycling, proliferation or differentiation, processes that are crucial to cell growth control. 

miRs-130a, 15a, 27b, and 106b ranked variably within the lists for stability, depending on the software used and the genotype or muscle groups analysed. Of note, miR-15a was consistently ranked highest by Bestkeeper, and typically scored in the top 5 regardless of grouping for all 3 reference finding programs, though often scored highest in ‘WT only' muscle groupings (miR-15a also showed a strong Bestkeeper performance in our primary miFinder panel). This miR has a key role in regulation of cell differentiation and maturation and plays a tumour suppressor role in cancer cell proliferation, differentiation and apoptosis ( Liu *et al.*, 2019).

On the basis of these results, miR-191, let-7b, miR-125a and miR-15a were considered as suitable candidates, with a normalisation factor derived from these (NF4) being selected for normalising miR expression in DE50-MD and WT dog muscle regardless of age, or muscle type. In contrast, miR-29b and snRNA U6 were the least stable RNAs by all 3 methods of analysis, with snRNA U6 scoring as the least stable of the 9 RNAs for all groupings analysed. snRNA U6 is an RNA located at the heart of the spliceosome, and has been widely used as a reference miR, with studies showing its stable expression in specific disease states and cell types ( Duan *et al.*, 2018; Han *et al.*, 2014). However, others have found that snRNA U6 expression showed high inter-individual variation within disease states ( Davoren *et al.*, 2008) and varied across healthy human tissue types ( Peltier & Latham, 2008). In further demonstration of reference RNA tissue specificity, we previously found that miR-223 was a suitable reference miR for DE50-MD and WT dog serum ( Riddell *et al.*, 2021), however in the current study miR-223 scored poorly for stability in skeletal muscle by Normfinder, geNorm and BestKeeper analysis, ranking 67 ^th^, 68 ^th^ and 71 ^st^ out of 73 respectively; this emphasises the need for identification of suitable miRs for each novel tissue analysed. Here, snRNA U6 scored poorly under essentially every comparative scenario tested, suggesting it is not a suitable reference RNA for miR analysis in WT or DE50-MD skeletal muscle: as shown in Figure 10, normalisation of this small RNA using our four-miR reference (NF4) revealed expression to be increased in dystrophic muscle, but also declining with age in both genotypes. Without specific confirmation, we suggest that it is likely inappropriate for use as a reference miR in skeletal muscle from other canine DMD models, and (arguably) in other species.

The second lowest ranking RNA was miR-29b. This miR was shortlisted on the basis of its strong Bestkeeper stability scores in our primary screen, though it did not perform as well under Bestkeeper analysis of WT samples alone; it was also not in the top 20 most stable genes for any of the groupings (all samples, WT-only or DE50-MD only) for either geNorm or Normfinder analyses. miR-29b is one of 3 miRs in the miR-29 family. All 3 miRs have tumour suppressor functions, and abnormal expression of miR-29 is associated with various cancers ( Yan *et al.*, 2015). In addition, miR-29b has a role in skeletal muscle atrophy ( Li *et al.*, 2017), a feature of the DMD phenotype that is evident in the DE50-MD dog model from 6-months of age ( Hornby *et al.*, 2021). miR-29b is elevated in rodent models of muscle atrophy caused by denervation, dexamethasone treatment, fasting and cancer cachexia, and transfection of C2C12 myotubes with miR-29b is necessary and sufficient to induce fibre atrophy *in vitro* ( Li *et al.*, 2017). However, miR-29 family members also have anti-fibrotic roles, with expression of both miR-29a and miR-29c decreased in DMD patient quadriceps muscle and myoblasts ( Zanotti *et al.*, 2015). Fibrosis is a prominent feature of DE50-MD skeletal muscles from as young as 3-months of age, and dystrophy-associated fibrotic accumulation is found in all the muscle groups analysed in the current study ( Hildyard *et al.*, 2022). Reference finder results for our extended panel of muscle samples (VL 3–18 months and 18-month CRT, SEM, TRI and DIA), suggest that miR-29b is not a suitable reference miR for DE50-MD or WT skeletal muscle. In fact, when normalised to NF4, miR-29b was significantly altered in DE50-MD compared to WT muscle ( Figure 9): in 3-month VL muscle, expression was higher in dystrophic tissue, but while levels subsequently increased with age in both genotypes, increases in healthy muscle were more profound: from 9 months onward, WT expression was greater than DE50-MD. A corollary of this is that at 6 months of age (the age-group analysed by miFinder), no difference was found between genotypes. This both explains the ranking of this miR in our primary screen and illustrates the importance of assessing candidates using broader sample panels. The behaviour of this miR agrees with previous findings of reduced expression of closely related miR-29a and miR-29c in DMD patient muscle ( Zanotti *et al.*, 2015) and with the fibrotic phenotype of DE50-MD muscle ( Hildyard *et al.*, 2022). Expression of collagen mRNA is elevated in DE50-MD compared to WT dogs at all ages, with greatest differences between genotypes occurring in older animals (12–18 months), where expression declines in healthy muscle but remains elevated in dystrophic. This is indicative of ongoing pathological collagen deposition/remodelling ( Hildyard *et al.*, 2022), and aligns well with concomitant reductions in expression of the anti-fibrotic miR-29b in these older DE50-MD animals. Expression of miR-29b in muscle might also correlate with the amount of contractile muscle tissue present in the analysed muscle biopsies; with advancing disease severity, functional muscle tissue becomes increasingly replaced by non-contractile tissues such as fibrosis and fibro-adipogenic precursor cells. If miR-29b is primarily muscle cell-derived and plays a key role in muscle atrophy, the progressive loss/replacement of muscle tissue might result in a decline in measured miR-29b in biopsies with increasing DE50-MD dog age.

Given the behaviour of snRNA U6 and miR-29b revealed following normalisation to NF4, we normalised each of our 4 reference miRs individually to this collective factor to assess their respective contributions. There was no significant effect of genotype for either miR-191 or let-7b, however miR-125a was increased very slightly (1.1-fold increase), while miR-15a was decreased to a similar extent (1.1-fold decrease) in DE50-MD compared to WT samples: these modest differences between genotypes are of equivalent but opposite sign, and can thus be essentially negated by taking the mean of the 2 miRs (though this would necessarily require both miR-125a and miR-15a to be used in combination rather than individually). Thus, miR-191 and let-7b could be used individually, in combination with each other, or in combination with both miR-125a and miR-15a to produce a normalisation factor for DE50-MD and WT muscle samples. The use of multiple reference RNAs is recommended for quantification of any target of interest ( Bustin *et al.*, 2009). Quantification of multiple validated reference miRs could help account for any impact that an experimental condition may have on an individual reference miR. For this reason, we proceeded with the geometric mean of all 4 validated reference miRs as our normalisation factor.

Finally, to evaluate our candidates in a therapeutic context we used NF4 to normalise expression of a panel of miRs of interest to the DE50-MD phenotype: dystromiRs miR-1, -133a and -206, and fibromiR miR-214. Expression of both miR-1 and miR-133a within our sample collection was lower in DE50-MD compared to WT: approximately 3-fold lower for miR-1 and 2-fold lower for miR-133a. Conversely, miR-206 did not significantly differ between genotypes. Reduced muscle miR-1 and miR-133a alongside unaltered miR-206 expression has previously been reported in muscle samples from DMD patients (extensor digitorum brevis muscle) compared to healthy control tissue ( Zaharieva *et al.*, 2013). Studies of *mdx* mice have also shown reduced or not significantly altered miR-1 and -133a expression, while miR-206 is upregulated by 3- to 10-fold (quadriceps, CRT, DIA, TRI and heart) ( Israeli *et al.*, 2016; Roberts *et al.*, 2012; Yuasa *et al.*, 2008). In contrast, an earlier study found increased expression of miR-206 in the *mdx* diaphragm, but not in the soleus and plantaris (pelvic limb) muscles ( McCarthy *et al.*, 2007). The CXMD _J_ dog model of DMD was shown to have reduced miR-206 in the CRT muscle at 3 separate timepoints, however this was based on a single animal ( Yuasa *et al.*, 2008). Some of these discrepancies in miR-206 expression between studies could be reasonably attributed to muscle group analysed, however the panel of muscles analysed in the DE50-MD dog largely overlapped with that used in the *mdx* mouse study by Roberts *et al.*, 2012. Another factor that could enhance or diminish differences between groups is choice of reference RNAs; the reference RNAs used previously varied study-to-study and included miR-16 ( Roberts *et al.*, 2012), miR-24 ( McCarthy *et al.*, 2007), miR-93 ( Israeli *et al.*, 2016) and snRNA U6 ( Yuasa *et al.*, 2008; Zaharieva *et al.*, 2013). Alternatively, species differences might account for the results, with perhaps mouse miR expression differing from the more phenotypically similar human DMD patients and DMD dog models. As suggested (above) for miR-29b expression in DE50-MD muscle samples, lower dystromiR expression might relate to loss of muscle tissue via atrophy/fibrosis: these features are prominent in dog DMD models and in human DMD patients, whereas the phenotypically mild *mdx* mouse exhibits minimal fibrosis, and muscle hypertrophy rather than atrophy. We have previously shown that DE50-MD dogs have markedly elevated dystromiRs in serum while they are essentially absent in WT dog serum, and that circulating creatine kinase (CK) activity positively correlates with relative quantity of all three dystromiRs ( Riddell *et al.*, 2021). CK is highly abundant in myofibres and studies of dystrophic muscle have concluded that elevations in serum CK activity are a result of micro-lesions to the sarcolemma as a result of muscle contraction that allow CK leakage ( Vilquin *et al.*, 1998). We have recently shown that circulating CK activity increases following a 6-minute walk test in the DE50-MD dog and that this exercise-induced elevation is correlated with serum quantity of all three dystromiRs ( Riddell *et al.,* 2024a). In combination with our findings of reduced or unaltered dystromiRs in DE50-MD muscle, this suggests that, like CK activity, serum dystromiRs are good biomarkers of muscle fibre integrity, rather than biomarkers of pathophysiological processes within dystrophic fibres.

miR-214, a so-called fibromiR due to its strong association with muscle fibrotic content in DMD patient muscle biopsies ( Arrighi *et al.*, 2021), was elevated in all DE50-MD muscles tested, at all time-points, by an average of 2-fold in VL muscle and 4-fold in 18-month old CRT, SEM, TRI and DIA. This miR showed a mild decrease in expression over time in both DE50-MD and WT muscle samples. Previous work, calculating fibrosis fraction of DE50-MD compared to WT muscle showed the same decreasing pattern of fibrosis fraction with age, that was concluded to result from growth-associated fibre hypertrophy and consequent decreasing muscle fibre endomysium per unit area of muscle ( Hildyard *et al.*, 2022): a similar phenomenon could explain the modest age-associated decrease seen in miR-214. Nonetheless, miR-214 expression is clearly elevated in dystrophic canine muscle, and thus represents a novel biomarker of the DE50-MD phenotype. This miR, alongside the anti-fibrotic miR-29b, could serve as a valuable marker for muscle fibrosis and remodelling in future pre-clinical trials involving this model. Future work, to investigate whether serum miR-214 levels correlate with muscle miR-214 expression, could potentially provide a non-invasive measure of muscle fibrosis, something that would be of marked benefit practically and ethically in our dogs and (if present) in patients.

To conclude, we have identified a panel of stable miRs that can be used to normalise microRNA expression in DE50-MD and WT control dog muscle: a normalisation factor derived from miR-191, let-7b, miR-125a and miR-15a appears to be a highly stable and robust representation of total miR content, regardless of genotype, age or muscle type. We note that while use of multiple references is essential for good data normalisation, as specified in the MIQE guidelines ( Bustin *et al.*, 2009), use of four might be considered excessive. Our data illustrates the strength of these four in deciphering even subtle alterations in expression patterns, but for investigators wishing to use a reduced panel, miR-191 and let-7b would serve as such. As we show here, miR-125a and miR-15a are also strong references, but should only be used in mutual combination, as they exhibit modest but consistent dystrophy-associated expression patterns, of equal but opposite sign.

We also show that the dystromiRs miR-1 and miR-133a are decreased in DE50-MD dogs, while miR-206 is not: this is similar to DMD patients, but is not seen in mouse, demonstrating the clinical utility of the DE50-MD model. We further show that miRs can be used to assess fibrosis: anti-fibrotic miR-29b is decreased and pro-fibrotic miR-214 is increased in DE50-MD compared to WT dogs. Finally, we reveal that snRNA U6 is unsuitable for normalisation in this model.

## Ethics and consent

The study was conducted within project license P9A1D1D6E (granted 11 June 2019) assigned under the Animal (Scientific Procedures) Act 1986, according to UK legislation and was approved by the Royal Veterinary College local Animal Welfare Ethical Review Body. Dogs were observed daily by animal technician staff and any concerns were reported to the Study director, Named Veterinary Surgeon and Named Animal Care and Welfare Officer. Humane endpoints were established before commencing the study, (see “Methods: Animal husbandry”). None of the dogs included in this study reached humane end-points during the 18-month study period.

## Data Availability

Figshare: Identification of reference microRNAs in skeletal muscle of a canine model of Duchenne muscular dystrophy. https://doi.org/10.6084/m9.figshare.25054877 ( Riddell *et al.*, 2024b). This project contains the following underlying data: miFinder panel – underlying data (Cq and melt curve results for full miFinder panel) miFinder panel – Cq and RQ summary underlying data (Cq and RQ calculations for full miFinder panel) miRCURY Assays – underlying long data (Cq and melt curve results for individual miRCURY assays) miRCURY Assays – Cq and RQ summary underlying data (Cq and RQ calculations for individual miRCURY assays) miRCURY Assays – cross plate calibrators (Cq corrections for cross plate calibrations) DystromiRs – underlying data (Cq, melt curve and RQ calculations for dystromiRs-1, -133a and -206) FibromiRs – underlying data (Cq, melt curve and RQ calculation for fibromiR-214) This project contains the following extended data: Extended data - miFinder panel full results (GeNorm M values, Bestkeeper Pearson correlations and Normfinder stability values for the full miFinder panel) Extended data – Author Checklist - E10 DE50-MD Reference miRs Extended data – BestKeeper 100 Data are available under the terms of the Creative Commons Attribution 4.0 International license (CC-BY 4.0).
