# Supplementary material for: Identification of reference microRNAs in skeletal muscle of a canine model of Duchenne muscular dystrophy
Source: Wellcome Open Res. 2024 Nov 20;9:362. Originally published 2024 Jul 10. [Version 2] doi: 10.12688/wellcomeopenres.22481.2 (PMC11621615; doi:10.12688/wellcomeopenres.22481.2)
Supplement: Peer review history

# Reviewer report: Reviewer response for version 2

**DOI:** 10.21956/wellcomeopenres.25798.r114657

**Published:** 2024-12-31

**Authors:** Liubov Gushchina

**Affiliations:** 1 Nationwide Children's Hospital, Columbus, USA

This manuscript is of great interest to clinicians and researchers studying any forms of muscular dystrophy and is especially important in the field of DMD. The study is very easy to read, well presented and discussed. I have a few very minor suggestions that I hope the authors will find useful.

 The author mentioned that an unpaired, two-tailed t-test was used to compare results between WT and DE50-MD animals. Did the authors use the Mann-Whitney test or the Kolmogorov-Smirnov test?

 I would recommend checking all the N values in the following figure legends: Figures 9, 10 and 11 as they show N=6 for the VL muscle. However, if we count the dots, which I assume represent individual samples, the number of samples would be somewhere between 3 and 6. If so, please explain why some samples were excluded from the analysis and how many samples were analyzed in each experiment.

 The authors have provided a very comprehensive work, but I have not seen whether the results of this work will be uploaded to any of the repositories. Please clarify, otherwise I would recommend publishing the raw data as part of the supplementary information.

Is the work clearly and accurately presented and does it cite the current literature?

Yes

If applicable, is the statistical analysis and its interpretation appropriate?

Yes

Are all the source data underlying the results available to ensure full reproducibility?

Yes

Is the study design appropriate and is the work technically sound?

Yes

Are the conclusions drawn adequately supported by the results?

Yes

Are sufficient details of methods and analysis provided to allow replication by others?

Yes

Reviewer Expertise:

Muscular dystrophies, non-coding RNAs, gene therapy development

I confirm that I have read this submission and believe that I have an appropriate level of expertise to confirm that it is of an acceptable scientific standard, however I have significant reservations, as outlined above.

# Reviewer report: Reviewer response for version 2

**DOI:** 10.21956/wellcomeopenres.25798.r112489

**Published:** 2024-12-05

**Authors:** Matthew Alexander

**Affiliations:** 1 The University of Alabama at Birmingham, Birmingham, Alabama, USA

I have no additional comments. The edits and responses to my original critiques are sufficient.

Is the work clearly and accurately presented and does it cite the current literature?

Yes

If applicable, is the statistical analysis and its interpretation appropriate?

Yes

Are all the source data underlying the results available to ensure full reproducibility?

Yes

Is the study design appropriate and is the work technically sound?

Yes

Are the conclusions drawn adequately supported by the results?

Yes

Are sufficient details of methods and analysis provided to allow replication by others?

Yes

Reviewer Expertise:

muscular dystrophies, animal models, zebrafish drug screening, non-coding RNAs (ncRNAs)

I confirm that I have read this submission and believe that I have an appropriate level of expertise to confirm that it is of an acceptable scientific standard.

# Reviewer report: Reviewer response for version 2

**DOI:** 10.21956/wellcomeopenres.25798.r112488

**Published:** 2024-11-25

**Authors:** Patryk Konieczny

**Affiliations:** 1 Adam Mickiewicz University, Poznań, Poland

The authors addressed my concerns and I have no more questions. Congratulations on the very nice work!

Is the work clearly and accurately presented and does it cite the current literature?

Yes

If applicable, is the statistical analysis and its interpretation appropriate?

Yes

Are all the source data underlying the results available to ensure full reproducibility?

Yes

Is the study design appropriate and is the work technically sound?

Yes

Are the conclusions drawn adequately supported by the results?

Yes

Are sufficient details of methods and analysis provided to allow replication by others?

Yes

Reviewer Expertise:

muscle regeneration, function of dystrophin isoforms, regulation of DMD gene expression

I confirm that I have read this submission and believe that I have an appropriate level of expertise to confirm that it is of an acceptable scientific standard.

# Reviewer report: Reviewer response for version 1

**DOI:** 10.21956/wellcomeopenres.24770.r94114

**Published:** 2024-09-03

**Authors:** Patryk Konieczny

**Affiliations:** 1 Adam Mickiewicz University, Poznań, Poland

The authors focus on finding the appropriate normalizing miRNAs that do not differ in abundance in various muscles and at different ages in wild-type and DE50-MD dogs. Since four such miRNAs were found, two of which could be used individually and the remaining two only in combination, the manuscript paves the way for future therapeutic studies, in which other miRs could be accurately assessed in various muscles. The study is methodically correct and very nicely presented and discussed. I have only minor suggestions that authors may find useful:

  

1. **Introduction:** the most convincing explanation to me would be that both muscle mass and muscle condition influence the miR amounts in serum. For example, steroid treatment suppresses the immune response and the collapse of muscle fibers and therefore, fewer molecules leak to the bloodstream. On the other hand, older and more affected patients have very low muscle mass, which would again result in fewer miRs in serum.
2. **Introduction:** based on the text, it is very difficult to conclude what is the advantage of checking miR in muscles/biopsies over serum. What are the advantages/disadvantages of these methods? Should both of them be used in conjunction upon testing of a therapeutic treatment?  
3. **Results:** A diagram delineating methodological approach taken by the authors could be included.
4. **Results/Discussion:** I am surprised that the authors did not test the selected miRs in serum. Could the same miRs be used as normalizing factors in serum?

Is the work clearly and accurately presented and does it cite the current literature?

Yes

If applicable, is the statistical analysis and its interpretation appropriate?

Yes

Are all the source data underlying the results available to ensure full reproducibility?

Yes

Is the study design appropriate and is the work technically sound?

Yes

Are the conclusions drawn adequately supported by the results?

Yes

Are sufficient details of methods and analysis provided to allow replication by others?

Yes

Reviewer Expertise:

muscle regeneration, function of dystrophin isoforms, regulation of DMD gene expression

I confirm that I have read this submission and believe that I have an appropriate level of expertise to confirm that it is of an acceptable scientific standard, however I have significant reservations, as outlined above.

# Response

**Published:** 2024-11-14

**Authors:** Dominique Riddell

**Affiliations:** Royal Veterinary College, London, UK

Thank you very much for your careful consideration of our article. We are happy to address your questions:

  

1. ***Introduction:** the most convincing explanation to me would be that both muscle mass and muscle condition influence the miR amounts in serum. For example, steroid treatment suppresses the immune response and the collapse of muscle fibers and therefore, fewer molecules leak to the bloodstream. On the other hand, older and more affected patients have very low muscle mass, which would again result in fewer miRs in serum.*
**Response: **We agree with your conclusions, it is likely a combination of these factors. We have edited our paragraph discussing serum dystromiRs (paragraph 2) to emphasise these points.
2. ***Introduction:** based on the text, it is very difficult to conclude what is the advantage of checking miR in muscles/biopsies over serum. What are the advantages/disadvantages of these methods? Should both of them be used in conjunction upon testing of a therapeutic treatment?  *
**Response: **We have now added a section in paragraph 4 of the introduction that details our views on the utility of quantifying miRs in muscles; we believe that there is a place for miR analysis in both serum and muscle – as each addresses somewhat different issues. In essence, the key benefit of the assessment of muscle expression of miRs is that it is not confounded by overall muscle mass or muscle fibre (sarcolemmal) integrity both of which of course are major factors in DMD. Muscle-specific miR results therefore offer mechanistic insight into processes occurring within a particular muscle and, in the case of a single biopsy sample, within tissue where direct histological assessment is possible – for example, measurement of fibromiR expression. That is not possible with the more global assessment via serum miR measurements that are confounded by muscle mass and by muscle fibre integrity. Indeed, notably (in accordance with the other reviewer’s comment), there are prominent differences in serum (our earlier work) vs muscle (current work) with relative expression of some miRs (dystromiRs 1, 133a and 206 in particular) which highlight the important contrasts between muscle miR analysis vs serum analysis.
3. ***Results:** A diagram delineating methodological approach taken by the authors could be included.*
**Response: **This is a great suggestion thank you. We have now added a diagram (Figure 2) showing the workflow and approach we took.
4. ***Results/Discussion:** I am surprised that the authors did not test the selected miRs in serum. Could the same miRs be used as normalizing factors in serum?*
**Response: **We have previously identified optimal reference miRs in DE50-MD canine serum, which were subsequently used to normalise DE50-MD serum dystromiR concentrations (Riddell D, Hildyard J, Harron R, et al.: Longitudinal assessment of bloodborne musculoskeletal disease biomarkers in the DE50-MD dog model of duchenne muscular dystrophy [version 2; peer review: 2 approved]. Wellcome Open Res. 2021; 6: 354.) . Indeed, identification of reference miRs was performed as pilot work for this study and led to the conclusion that miR-223 was expressed stably and robustly across all serum samples tested and hence was a suitable reference gene. Whilst our earlier study is referred to in the text, we did not clearly state that a DE50-MD model serum reference miR had previously been established and so now refer to our previous work in both the introduction (paragraph 6) and discussion (paragraph 8). Interestingly, miR-223 scored very poorly in our muscle samples in the current study by Normfinder, geNorm and Bestkeeper analysis, ranking 67 ^th^, 68 ^th^ and 71 ^st^ out of 73 candidates respectively, despite being suitable for serum. This therefore concurs with our response to your previous point about the relative merit of serum vs muscle miR analysis.

# Reviewer report: Reviewer response for version 1

**DOI:** 10.21956/wellcomeopenres.24770.r94110

**Published:** 2024-09-02

**Authors:** Matthew Alexander

**Affiliations:** 1 The University of Alabama at Birmingham, Birmingham, Alabama, USA

The manuscript is very straight forward and the experiments are easy to follow. Data presentation is standard and statistical analysis is appropriate. I think that I would recommend the additional citations for: Sanson et al., Sci. Reports, 2020; and Nghiem et al., Am. J. Pathol., 2013 that are missing. Both studies highlight microRNAs that are dysregulated in canine models of DMD. I think that the authors could consider expanding a bit more on why specific "dystromiRs/myomiRs" are not as disrupted in their models/expression analysis. Notably, the miR-206 showing no significant differences between DE-50 and WT dogs. One explanation might be degree of severity of the pathology of the dogs taken at this stage compared with mouse models and human biopsies. miR-146a also changes in expression in mouse and human models of DMD/BMD and appears to be listed in the "Most Stable" category for Table 2. Please clarify. Otherwise, I think the manuscript is appropriate and would be an excellent addition to the DMD animal modeling field.

Is the work clearly and accurately presented and does it cite the current literature?

Yes

If applicable, is the statistical analysis and its interpretation appropriate?

Yes

Are all the source data underlying the results available to ensure full reproducibility?

Yes

Is the study design appropriate and is the work technically sound?

Yes

Are the conclusions drawn adequately supported by the results?

Yes

Are sufficient details of methods and analysis provided to allow replication by others?

Yes

Reviewer Expertise:

muscular dystrophies, animal models, zebrafish drug screening, non-coding RNAs (ncRNAs)

I confirm that I have read this submission and believe that I have an appropriate level of expertise to confirm that it is of an acceptable scientific standard, however I have significant reservations, as outlined above.

# Response

**Published:** 2024-11-14

**Authors:** Dominique Riddell

**Affiliations:** Royal Veterinary College, London, UK

Thank you for your comments and supportive assessment of our work, we are glad to answer the questions raised:

1. *I think that I would recommend the additional citations for: Sanson et al., Sci. Reports, 2020; and Nghiem et al., Am. J. Pathol., 2013 that are missing. Both studies highlight microRNAs that are dysregulated in canine models of DMD.*
**Response: **Thank you for highlighting these studies, we have now referenced them in two relevant paragraphs (paragraphs 5 and 6) of the introduction.
2. *I think that the authors could consider expanding a bit more on why specific "dystromiRs/myomiRs" are not as disrupted in their models/expression analysis.* *Notably, the miR-206 showing no significant differences between DE-50 and WT dogs. One explanation might be degree of severity of the pathology of the dogs taken at this stage compared with mouse models and human biopsies.* **Response: **We are happy to expand on our list of possible explanations for the lack of disruption in miR-206 in paragraph 11 of the discussion. In fact, since submission of this article, we have published another study investigating the influence of exercise upon dystromiR levels in the blood, which we feel add further clarity to the dystromiR profile of DE50-MD dogs. We have previously shown that dystromiRs in DE50-MD serum correlate positively with CK activity (Riddell *et al*. 2021), and have since demonstrated that serum dystromiRs levels also correlate with a rise in circulating CK activity following exercise. CK is abundant in healthy muscle, as are the dystromiRs, and it has been concluded that its elevation in serum of dystrophic individuals is a consequence of microdamage to the sarcolemma that occurs during muscle contraction. Our findings of lowered or unchanged muscle dystromiR quantity in combination with elevated serum levels and correlation with circulating CK activity, thus support the theory that dystromiR dysregulation is a consequence of leakage into the circulation, rather than a primary pathophysiological process within muscle fibres. Indeed, lower quantities of certain dystromiRs within muscle might be precisely because of their leakage from muscle into the extracellular space in our opinion.
3. *miR-146a also changes in expression in mouse and human models of DMD/BMD and appears to be listed in the "Most Stable" category for Table 2. Please clarify.*
**Response: **We do not list miR-146a in table 2, however miR-146b is listed as one of the top 20 most-stable miRs in WT animals only, meaning that within this genotype miR146b expression was relatively stable across the 4 WT dogs tested. This miR was not however in the top 20 for DE50-MD dogs or when both genotypes were analysed together. Increased expression of miR-146b has been found in both DMD and BMD patient muscle (Eisenberg et al., 2007), as well as *mdx *mouse and GRMD muscle (Fiorillo et al., 2015). In agreement with this, for the miFinder panel (N=4) DE50-MD dog miR-146b expression was 16-fold higher than WT dog expression (See Supplementary data – Cq and RQ summary) and it ranked 73 ^rd^ , 72 ^nd^ and 73 ^rd^ out of 73 candidates for stability when analysing genotypes together by geNorm, Bestkeeper and Normfinder respectively. miR-146a expression, which is indeed elevated in DMD and BMD patients (McCormack et al., 2023), was 2-fold higher in DE50-MD compared to WT dogs and ranked 63 ^rd^, 65 ^th^ and 61 ^st^ by geNorm, Bestkeeper and Normfinder respectively. So, while miR-146b appears to be a relatively stable candidate in WT dog muscle (by Bestkeeper analysis only) it did not score highly across any other algorithm and in fact scored very poorly across all three reference finder software packages when genotypes were analysed together.
*Otherwise, I think the manuscript is appropriate and would be an excellent addition to the DMD animal modeling field.* Thank you for your supportive comment.
